# Over 200,000 kilometers of free-flowing river habitat in Europe is altered due to impoundments

**DOI:** 10.1038/s41467-023-40922-6

**Published:** 2023-10-09

**Authors:** Piotr Parasiewicz, Kamila Belka, Małgorzata Łapińska, Karol Ławniczak, Paweł Prus, Mikołaj Adamczyk, Paweł Buras, Jacek Szlakowski, Zbigniew Kaczkowski, Kinga Krauze, Joanna O’Keeffe, Katarzyna Suska, Janusz Ligięza, Andreas Melcher, Jesse O’Hanley, Kim Birnie-Gauvin, Kim Aarestrup, Peter E. Jones, Joshua Jones, Carlos Garcia de Leaniz, Jeroen S. Tummers, Sofia Consuegra, Paul Kemp, Hannah Schwedhelm, Zbigniew Popek, Gilles Segura, Sergio Vallesi, Maciej Zalewski, Wiesław Wiśniewolski

**Affiliations:** 1 National Inland Fisheries Research Institute, Olsztyn, Poland; 2 European Regional Centre for Ecohydrology of the Polish Academy of Sciences, Łódź, Poland; 3 University of Lodz, Łódź, Poland; 4 University of Natural Resources and Life Sciences, Vienna, Austria; 5 University of Kent, Canterbury, UK; 6 Technical University of Denmark, Silkeborg, Denmark; 7 Swansea University, Swansea, UK; 8 Durham University, Durham, UK; 9 RAVON, Nijmegen, The Netherlands; 10 University of Southampton, Southampton, UK; 11 Technical University of Munich, München, Germany; 12 Warsaw University of Life Sciences, Warszawa, Poland; 13 IS Environnement, Saint-Jean, France; 14 Hydronexus, Bologna, Italy

**Keywords:** Environmental impact, Ecological modelling

## Abstract

European rivers are disconnected by more than one million man-made barriers that physically limit aquatic species migration and contribute to modification of freshwater habitats. Here, a Conceptual Habitat Alteration Model for Ponding is developed to aid in evaluating the effects of impoundments on fish habitats. Fish communities present in rivers with low human impact and their broad environmental settings enable classification of European rivers into 15 macrohabitat types. These classifications, together with the estimated fish sensitivity to alteration of their habitat are used for assessing the impacts of six main barrier types (dams, weirs, sluices, culverts, fords, and ramps). Our results indicate that over 200,000 km or 10% of previously free-flowing river habitat has been altered due to impoundments. Although they appear less frequently, dams, weirs and sluices cause much more habitat alteration than the other types. Their impact is regionally diverse, which is a function of barrier height, type and density, as well as biogeographical location. This work allows us to foresee what potential environmental gain or loss can be expected with planned barrier management actions in rivers, and to prioritize management actions.

## Abstract (web summary)

European rivers have over a million barriers hindering aquatic species migration and altering freshwater habitats. This study quantifies the spatial extent of upstream fish habitat alteration caused by physical blockage and shows that impoundments have altered 10% or 200,000 km of free-flowing river habitat in Europe.

## Introduction

One of the main causes of biodiversity loss in the aquatic environment is the fragmentation of habitats by barriers^1^. A recently published pan-European-barrier inventory estimates that >1.2 million barriers of varying type and size are located across the European continent^2^, with 13% deemed legacy structures having little socio-economic value but causing potential ecological harm^3^. It is estimated that there are on average 0.74 barriers per river kilometer in Europe, with a median distance of 108 m between adjacent barriers^2^. Hydromorphological modification is the primary reason why 40% of European rivers and floodplains fail to achieve ‘good ecological status’^4^, based on standards of the European Union Water Framework Directive (EU WFD)^5^. A chief cause of this is the presence of artificial barriers (comprising 24% of all reported hydromorphological pressures^6^), which can drastically alter upstream and downstream river morphology, ecohydrology characteristics, and in-stream habitat quality as well as availability^7,8^. Although barriers are expected to offer a series of societal benefits, river fragmentation caused by man-made barriers can also negatively impact the sustainability of freshwater ecosystems^2,9,10^. Barriers affect freshwater ecosystems ability to support a wide array of ecosystem functions and services including maintenance of natural habitats, food provision, nutrient cycling, and flow regulation^11^.

Backwater impoundments created by artificial barriers alter upstream river hydraulics, creating pond-like environments that favor generalist and limnophilic species. Barriers also cause downstream changes in channel form (e.g. due to substrate depletion), flow hydrodynamics, sediment transport, water temperature, chemistry, and biology. The magnitude and extent of downstream habitat alteration is a consequence of upstream ponding-related modification of the local river hydraulics and biology and, therefore, difficult to quantify accurately. Ponding can be considered a source of habitat discontinuity which alters the species composition up- and downstream of a barrier^8^. Habitats shape the biodiversity, biomass, and bioproductivity of rivers, facilitating creation of ecological processes that strongly regulate river ecosystems^12,13^. Accordingly, there is ample evidence that the habitat mosaics formed in different areas are associated with a unique community structure of aquatic fauna and flora^14,15^. Habitat mosaics are influenced by macroscale landscape attributes, such as ‘high-level’ drivers of stream habitat evolution: geology, hydrology, and biology^16^. These drivers operate through derivative proxies like catchment topography, rainfall-runoff relationships, valley slope and valley confinement, sediment transport regime, channel boundary characteristics, vegetation^16^. Also, recently the human impact is become increasingly profound in terms of shaping habitats and ecosystems either directly or indirectly.

The cumulative impact of barriers on freshwater aquatic communities has not been fully documented nor are there appropriate tools to measure it, despite the dramatic loss of free-flowing large and medium-sized rivers^17^. Impoundments have been shown to have an array of impacts on worldwide biodiversity. Yet, little is known about the overall spatial extent of the biological impact of ponding in Europe. Fish are widely considered indicator species for assessing freshwater habitat alteration^18–20^. There are 381 species of freshwater fish inhabiting different biogeographic regions across Europe^21^. Although different ecological strategies exist within taxa, still many use similar habitats despite being separated without genetic exchange. We expect that fish communities consist of guilds with varying sensitivity to impoundment, while different barrier types have varying impacts depending on the macrohabitat area in which they are located. As a consequence, impounding impacts are expected to be barrier and geographically-specific.

**Fig. 1:**
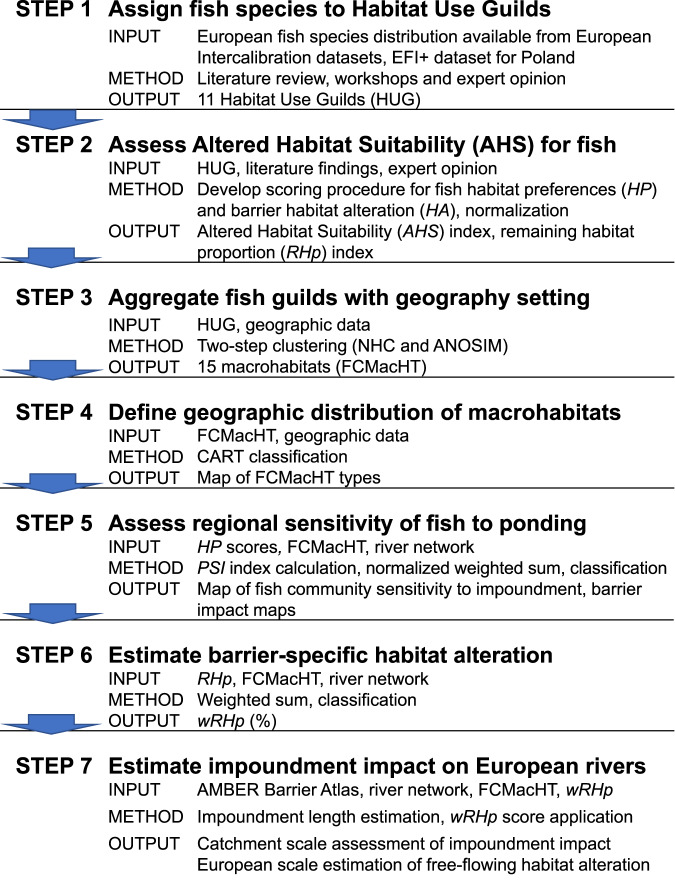
Conceptual Habitat Alteration Model for Ponding (CHAMP). Steps involved in the model building process.

In this work, we quantify the spatial extent of upstream fish habitat alteration caused by the physical blockage of free-flowing rivers. A Conceptual Habitat Alteration Model for Ponding (CHAMP) quantifies free-flowing river habitat left unaltered after human intervention (barrier placement). The general overview of steps undertaken to create the model is presented in Fig. 1 and described below (see Methods for details). It estimates the proportion of habitats altered by different barrier types located in different environmental settings from a point of view of fish communities expected there. Finally, using the existing barrier inventory^2^, we estimate the total river length that has been modified by man-made barriers. We estimate a conservative number of over 200,000 km or 10% of river length being altered due to impoundments in Europe and indicate a strategy for prioritization of the management actions. Furthermore, our classification of macrohabitats (FCMacHT) provides a consistent natural riverine habitat taxonomy for Europe. We conclude that it is not only barrier density that matters for biodiversity loss, but also the structure type and its location that determines the expected fish habitat structure and its potential alteration. The model can aid in identifying strategies for river management actions, such as dam removals or constructions, adaptations, channel restorations or regulations, and scenarios to reduce the impact.

## Results

**Fig. 2:**
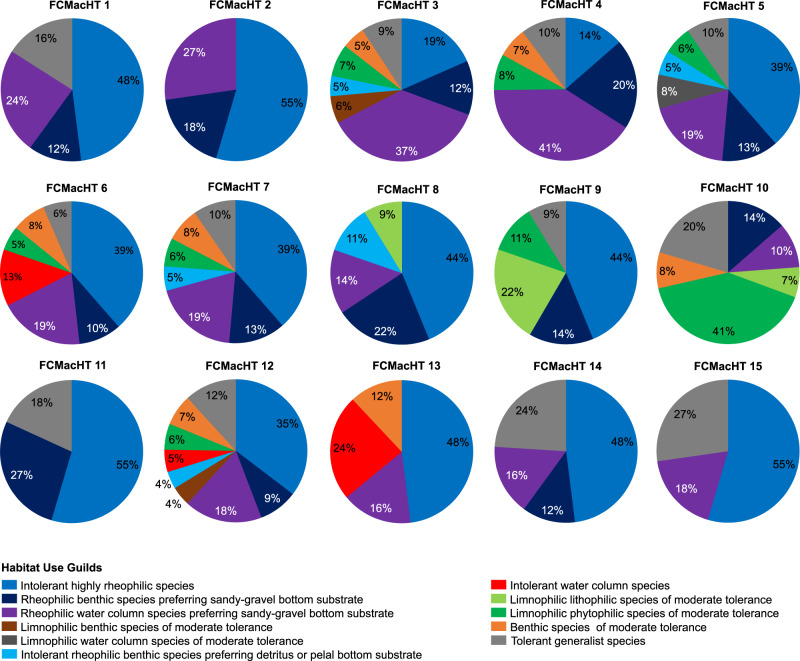
Structure of expected fish communities presented as a proportion of 11 Habitat Use Guilds for each FCMacHT. Guilds are ordered from more rheophilic/less tolerant to generalist/more tolerant species^68^. For FCMacHT descriptors, refer to Fig. 3 and Supplementary Information, Box 2.

**Fig. 3:**
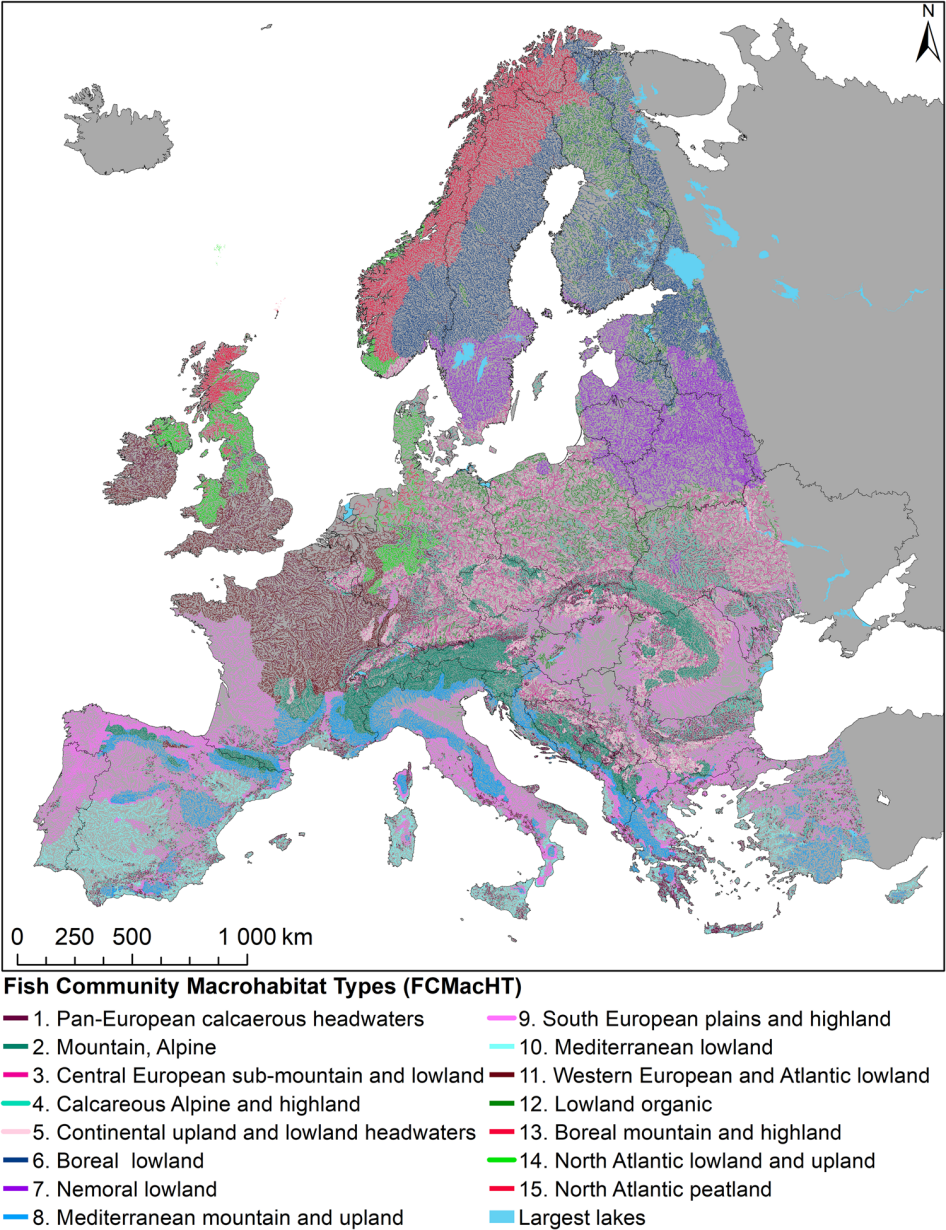
Fish Community Macrohabitat Types (FCMacHT) in European running waters. Data source: CCM v2.1^43^ for river segments, ECRINS^69^ for country borders.

Following the fact that many species share the same habitats and assuming that they also share a common sensitivity to habitat alteration European fish species were assigned to habitat use guilds (HUGs), which served as indicators of barrier impacts. Therefore, regionally occurring guild compositions and broad environmental characteristics, such as river slope, stream order, catchment size, altitude, catchment geology, and bioclimatic zone (Supplementary Figure 1), were grouped into 15 Fish Community Macrohabitat Types (FCMacHT, Fig. 2). They define expected proportions of habitats for each HUG at a river segment scale depending on its geography and hydromorphology. The result of extrapolation of fish community structure and their habitat’s environmental setting of near-natural sites is a map of the distribution of fifteen macrohabitat types (FCMacHTs) over Europe (Fig. 3) uniquely characterized by different proportions of HUGs.

**Fig. 4:**
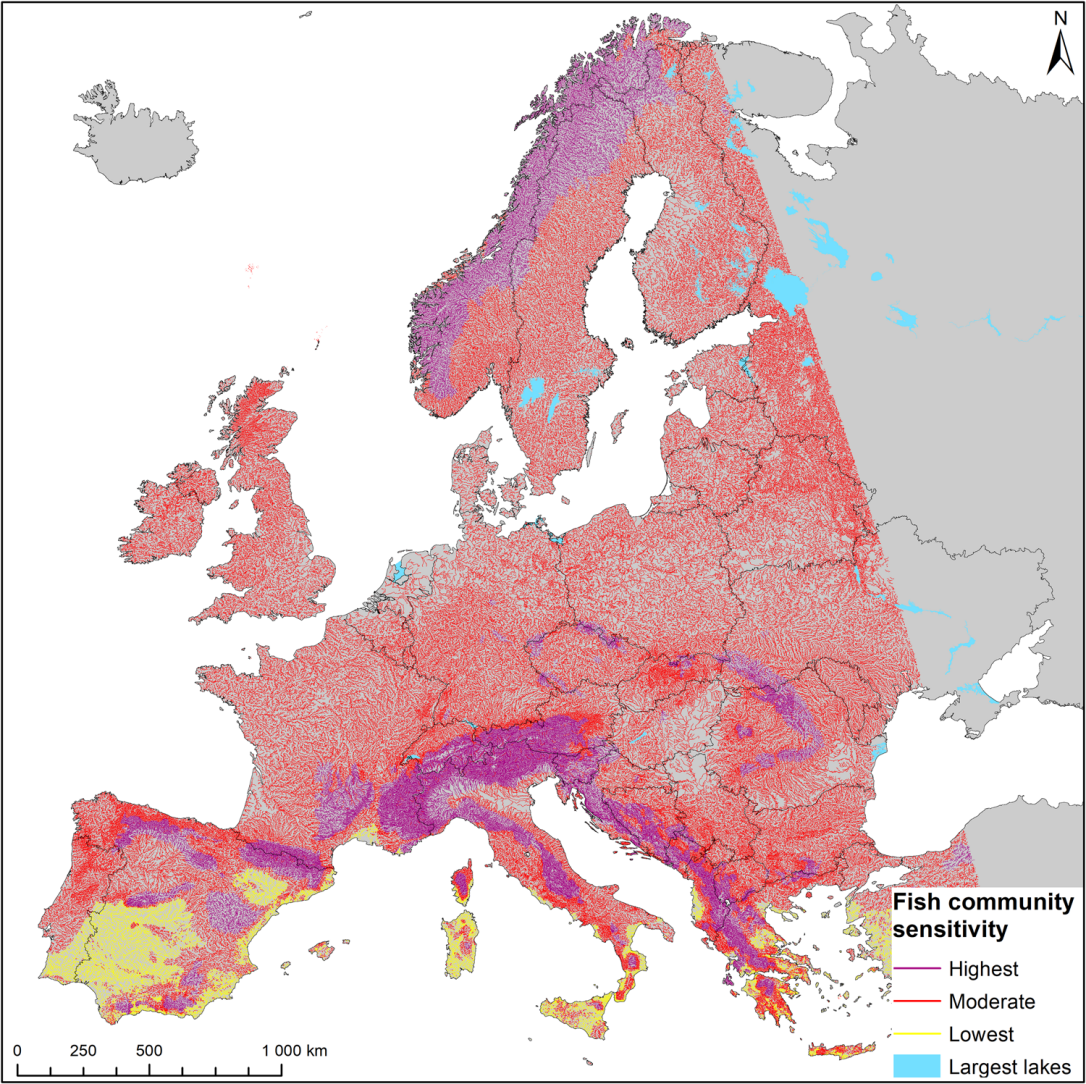
Fish community sensitivity to impounding. Highest sensitivity: fish communities where >55% of preferred habitat characteristics may potentially be affected by in-river barriers and Lowest if less than 45% of those are. Moderate sensitivity is for the values in between. It is calculated as a sum of Habitat Preferences weighted by guild proportions in the fish community over the maximum. The threshold classification method is Jenks Natural Breaks^70^.

**Fig. 5:**
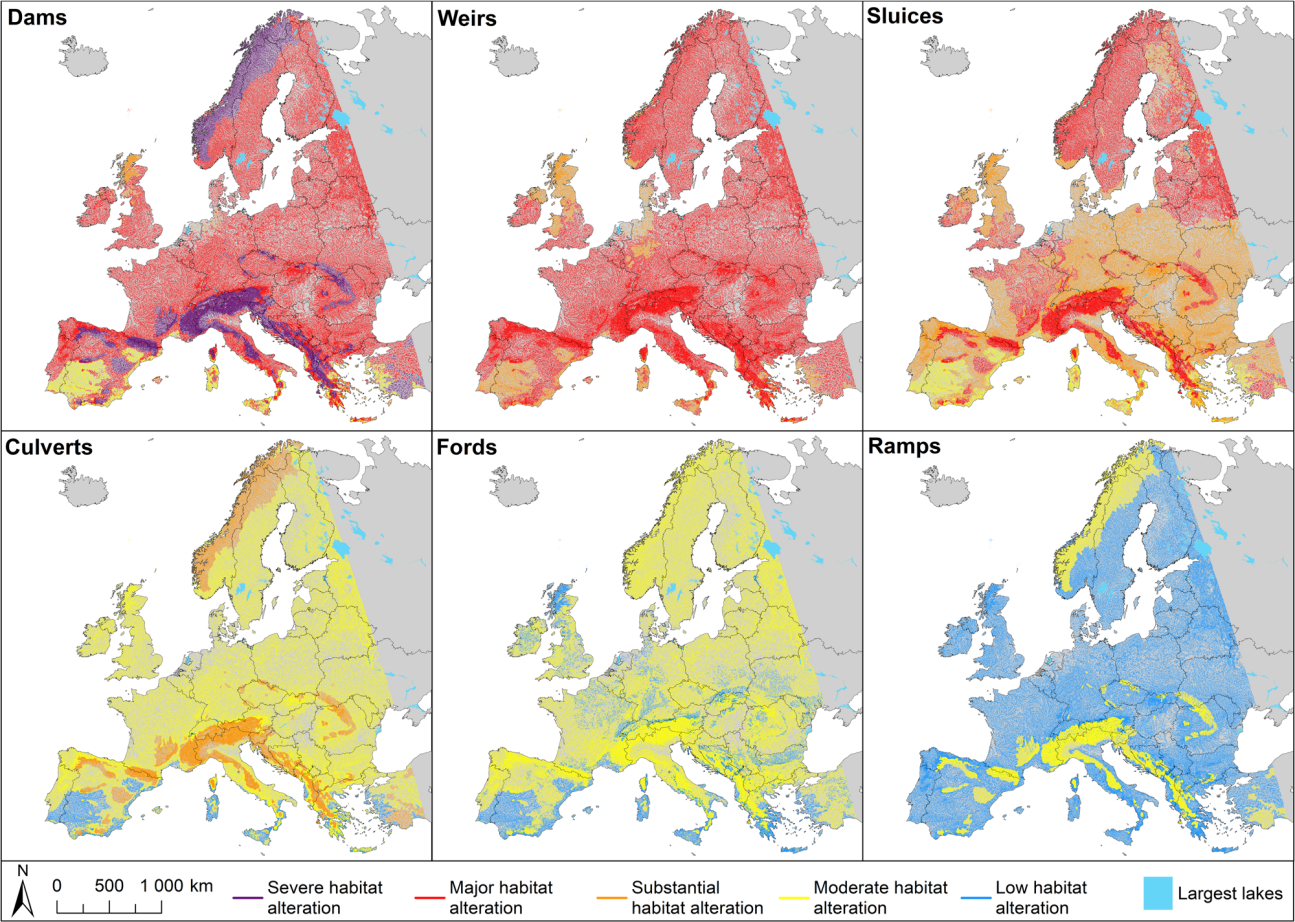
Estimated barrier habitat impact across European rivers with respect to barrier type. Results can help inform where the building of new structures is likely to cause large habitat alteration or where restoration measures are likely to produce the largest habitat gains. Each panel refers to an individual barrier type. Classification based on *wRHp* values. Purple—severe habitat alteration (≤25%), red—major habitat alteration (26–50%), orange—substantial habitat alteration (51–75%), yellow—moderate habitat alteration (76–90%), blue—low habitat alteration (>90%).

Further on, fish communities constituting guilds at each macrohabitat served as indicators of aggregated fish community sensitivity to impoundment across Europe (Fig. 4) caused by different barrier types (Fig. 5). High-sensitivity rivers are located in mountainous areas all over Europe, especially in Scandinavia, the Alps and Dolomites, Carpathian mountains and Iberian Peninsula mountains. These constitute a total of almost 450,000 km of river length that are anticipated to be the most vulnerable to ponding. These are followed by almost 1,500,000 km of river length in the rest of the continent, showing lower vulnerability. The lowest sensitivity to impoundment is attributed to some types of rivers in the Mediterranean region and Iberian Peninsula of just 100,000 km river length. It has to be noted that at this point, the obtained information on predicted fish community sensitivity to impounding does not account for the density of barriers nor other anthropogenic impacts that might be significant.

The impact of a barrier was defined as weighted Riverine Habitat proportion (*wRHp*) indicating a proportion of the remaining free-flowing habitat. It is a weighted sum of the remaining riverine habitat for a guild (*RHp*) and guild proportion in a given location. The lowest *wRHp* values indicating severe to substantial habitat alteration are associated with dams and weirs, followed by sluices (Table 1, Fig. 5). The dams alter river habitats the most in mountainous regions of southern Europe, the Alps, and Scandinavia, causing severe habitat alteration (<15% *wRHp*). A lower degree of impact is expected to occur for culverts, fords, and ramps, with prevailing moderate to low habitat alteration in the Mediterranean and the Iberian Peninsula (>73% *wRHp*). Overall, culverts, fords, and ramps show the highest *wRHp* values (>75% *wRHp*), meaning the greatest proportion of habitat unmodified. This leads to the conclusion that impounding impacts are expected to be barrier and geography-specific.

**Table 1:**
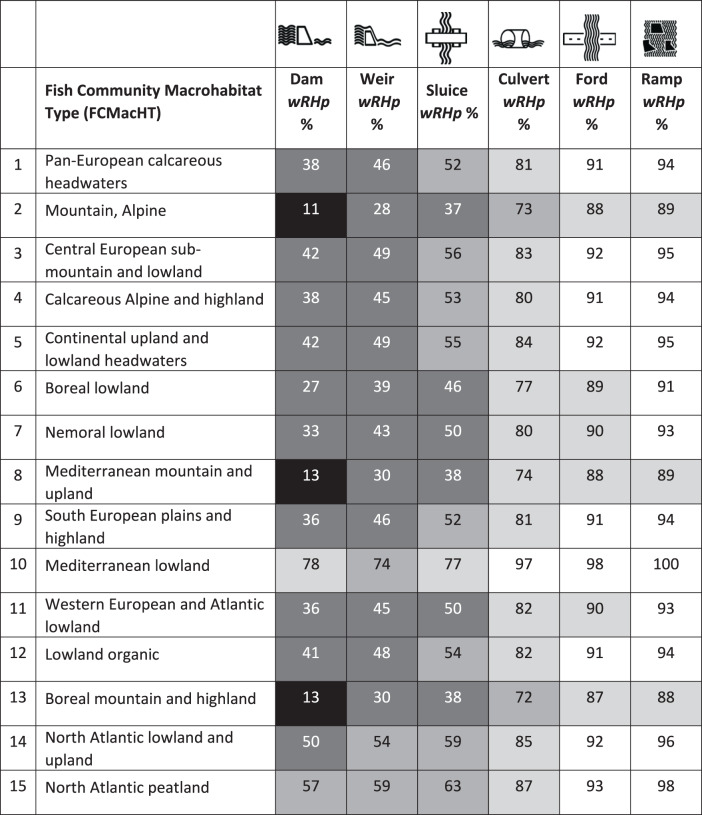
Weighted Riverine Habitat proportion (wRHp) with regard to barrier type and FCMacHT

Black—severe habitat alteration (≤25%), dark gray—major habitat alteration (26–50%), gray—substantial habitat alteration (51–75%), light gray—moderate habitat alteration (76–90%), white—low habitat alteration (>90%).

**Fig. 6:**
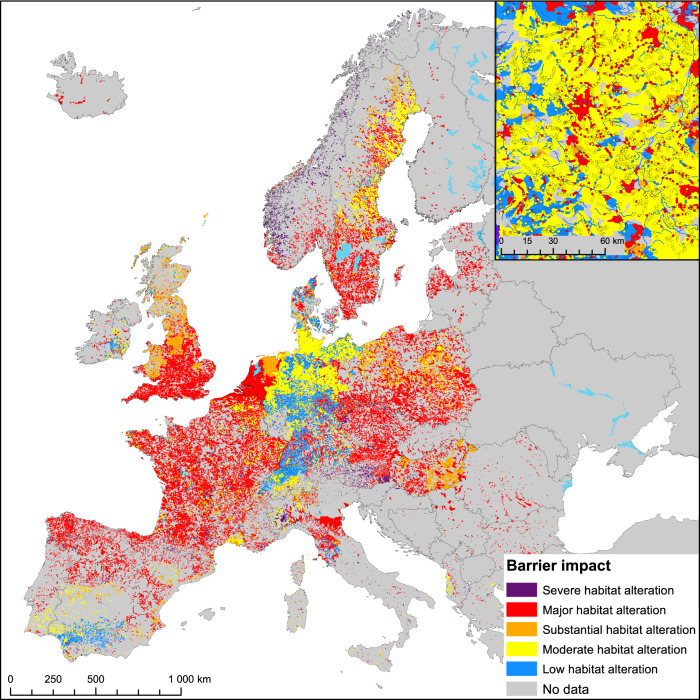
Projected barrier-level impounding impacts in European catchments. Median value of weighted riverine habitat proportion (*wRHp*) of barriers in the same catchment (refer to Table 1) was used. The inset figure’s extent is marked with a rectangle in the main map. Points in the inset figure represent individual recorded barriers. Regions with low per-barrier impact in Central Europe indicate a relatively high number of small barriers (culverts, fords, and ramps) with low individual impact contained in national registers, while major per-barrier impact in other parts of Europe indicates the prevalence of a relatively high number of large barriers and sensitive fish communities. Data sources: AMBER Barrier Atlas (acc. Nov. 2020^22^) for barrier locations, CCM v2.1^43^ for river segments and catchments.

These findings allowed us to project free-flowing river habitat alterations across Europe based on barriers recorded in the AMBER Barrier Atlas^22^ that served as a reference point. Each barrier was attributed with its FCMacHT and had the remaining free-flowing river habitat calculated. A prevailing *wRHp* from all barriers was derived at the catchment scale (Fig. 6).

From the estimated total impoundment length of just over 67,000 km, ca. 30,100 km (45%) still provides free-flowing river habitat (based on *wRHp*). This can be translated to a net alteration of approximately 36,900 km (55%) of river habitat known to be impacted (Table 2). However, the estimation of altered habitat length does not include all barriers but only those with height value available (64% of recorded dams, weirs, and sluices are missing such information). The significant underreporting of barriers (61% overall) within the AMBER Barrier Atlas^2^ needs to be also accounted for. Correcting for these two factors by extrapolation, allowed us to conclude that no less than 203,100 km of river habitat in Europe has been lost due to barrier impounding (Table 2). This conservative approximation represents almost 10% of the entire European river network. The true amount of river habitat alteration is likely much greater. Breaking it down, over 74% of habitat alteration (~151,000 km) is caused by weirs due to their high projected numbers (*n* = 441,829) relative to both dams and sluices (*n* = 71,010 and *n* = 24,940, respectively). By contrast, dams account for ~16% of habitat alteration (~33,400 km) and sluices a little over 9% (~18,800 km). On a per-barrier basis, severe habitat alteration is mainly concentrated in northwestern parts of Scandinavia and southern portions of Austria (Fig. 6), though this may caused by relatively uniform barrier data (only large barriers reported) in national barrier inventories.

**Table 2:** Estimated habitat alteration due to impounding from dams, weirs, and sluices

Barrier type	Number of barriers with height info*	Calculated impoundment length (km)	Calculated remaining habitat** (km)	Calculated habitat alteration (km)	Proportion of barriers with height info (%)	Underreporting error (%)	Est. impoundment length (km)	Est. remaining habitat length (km)	Est. habitat alteration (km)
Dam	28,913	22,180	8591	13,588	48.3%	15.7%	54,473	21,100	33,373
Weir	59,099	38,195	18,000	20,196	32.0%	58.2%	285,552	134,566	150,986
Sluice	4155	6686	3553	3132	59.5%	72.0%	40,129	21,328	18,801
Total	92,167	67,061	30,144	36,917	-	-	380,154	176,994	203,160

Data sources: AMBER Barrier Atlas (acc. Nov. 2020)^22^ for barrier locations and heights, barrier underreporting error from Belletti et al.^2^

^*^Excluded data with zero slope value.

**This is the remaining habitat of the impounded sections of the rivers estimated based on wRHp and FCMacHT.

## Discussion

There are over 600 thousand in-river barriers recorded in the AMBER Barrier Atlas^22^, though modeling suggests there may be more than 1.2 million barriers within Europe^2^. Information on barrier height is available for only 38% of recorded dams, weirs, and sluices. With this data it was estimated, conservatively, that over 203,100 km of free-flowing river habitat has been altered by barrier impounding. Major habitat alteration occurs throughout most of England, France Northwestern Europe, northern parts of Spain and Italy, and much of Eastern Europe. In contrast, rivers in the Balkans remain largely free-flowing, though currently, they face increasing threats from dam building and other anthropogenic pressures^2^. These rivers would benefit from explicit protection to shield them against future fragmentation.

Although the calculated altered habitat length equals to 10% of the total length of rivers in Europe, it needs to be recognized that this is equivalent to the combined length of all the rivers in, for example, Italy. Since, as mentioned before, impounding is only one aspect of impact to the biodiversity of the barriers, the sum total of all habitat alteration up- and downstream will be much greater. Moreover, this broad-scale study was limited to only assessing fish habitat, without taking into account the broader impact on the biodiversity (e.g. population fragmentation and genetic structure changes^23^) or locally specific boundary conditions. Another significant limitation is that habitat conditions investigated here are only those of adult, rearing, and growth life stages, which is probably the most resilient form of fish life. Life stages such as larval or spawning utilize usually narrower niches with specific requirements (like spawning substrate size), which are critical for biological processes. So, even if rearing and growth habitat is available, lack of habitat for other life stages may conflict with population conservation efforts for river fish fauna. Therefore, we can safely conclude that man-made barriers have profound impacts on fish habitat in European rivers.

This conclusion justifies one of the main goals of EU 2030 Biodiversity Strategy to create at least 25,000 km of free-flowing rivers^24^. By pointing out that the impact of impoundments strongly depends on their biogeographic location, this study fills one of the knowledge gaps for prioritization of barrier removals called for in the Guidance on Barrier Removal for River Restoration^25^.

With the model presented here, we document that impoundments from culverts, ramps, and fords result in low to moderate habitat alteration. In contrast impounding from dams, weirs, and sluices cause substantial to severe habitat alteration across most of the continent, except parts of the Mediterranean with its distinctive types of fish communities (Fig. 4 and Fig. 5). This is not to say that smaller structures like culverts, fords, and ramps have little impact. Due to their much greater prevalence within the landscape^2^, they disproportionately fragment rivers and streams, and obstruct aquatic organism dispersal, potentially leading to restricted range sizes, altered population structures, reduced spawning and recruitment success, genetic isolation, and local extinction^26,27^. We, therefore, suggest that an effective management prioritization procedure needs to factor in not only fish community habitat structure and sensitivity to impounding (Fig. 4), but also barrier density and location within river networks and the resulting influence on habitat accessibility.

A second key finding (confirming observations of other scientists^28,29^) is that low-head impoundment weirs located in mountainous areas may actually be more detrimental than impoundment weirs in lowlands areas. This is due to their more profound alteration of river hydraulics and the greater prevalence of habitat alteration suffered by rheophilic species in alpine areas. On top of this, there is a higher density of small dams in mountainous areas compared to large dams in lowland areas, thus potentially causing cumulatively greater river habitat alteration. Yet, the impoundments are usually shorter in the mountainous areas and that could reduce the cumulative impact.

Like in many other large-scale river fragmentation studies^2,17^, several shortcomings must be taken into account when interpreting the estimated extent of habitat loss caused by artificial barriers. For example, our assessment of barrier impacts on habitat availability was based on a literature review and expert judgment, but such knowledge is rather limited for most fish species, while barrier impacts and fish habitat preferences vary with water temperature, life stage, biophysical characteristics, and season^30,31^. These detailed aspects were not taken into account in our study. Likewise, barrier data was incomplete, and key drivers of barrier impacts such as barrier height were often missing, while no provision was made for natural fish barriers, as these are seldom mapped. Our assessment of the extent of fish habitat types and relative distribution of fish communities was based on regional calibration exercises scaled up across Europe. Finally, the diversity of ecological strategies for ecological change is taken into account partially by applying the generalized HUG concept. Aspects such as sedentary or opportunistic behavior are not considered. This can introduce additional inaccuracies. Clearly, more complete and detailed barrier and biological data, as well as novel barrier prioritization tools capable of accounting for data uncertainty^32^, would go a long way towards improving the assessment of barrier impacts and river restoration planning. This is especially important in relation to low-head structures, such as culverts, fords, and ramps, which are the most numerous^2^.

**Fig. 7:**
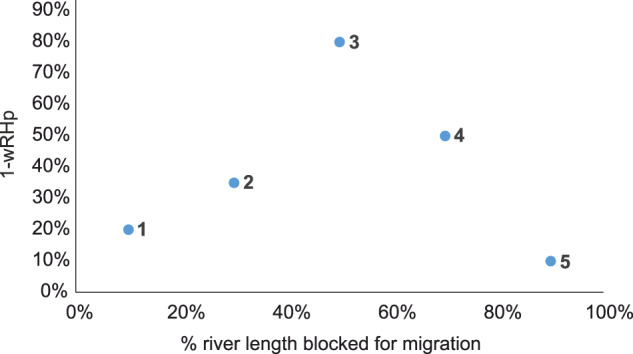
Hypothetical example of application of our results. We adapted restoration alternatives analysis to demonstrate the impact of five barriers in a watershed located on the main stem of the river. It demonstrates how much each barrier alters the free-flowing riverine habitat (1-wRHp) and how much of the upstream river length is no longer available for migrating fish. Barrier 5 is the closest to the river mouth.

This study provides the basis for further assessments of barrier impacts and reveals the substantial extent of impounded waters. Even at the current coarse level of accuracy, it offers a direction for developing strategies to enhance river biodiversity by reducing habitat alteration while differentiating between barrier types. For example, the results of this work could be used for prioritization of dam removals in a watershed by being incorporated into the Restoration Alternative Analysis model^33^. Figure 7 demonstrates a hypothetical scenario where altered habitat proportion is plotted against upstream river length blocked by 5 barriers downstream. By observing the Euclidian distance of the barrier indicators from the graph intercept we could conclude that although barrier 5, which is the closest to the river mouth requires only a well-functioning fish passage as it does not affect much the free-flowing river habitat. Barrier 1 located close to headwater and altering only small portion of habitat does not need a lot of attention. In contrast barrier 3, which introduces the greatest habitat alteration, requires more substantial mitigation actions that may include also barrier removal. Although improving fish passage offers one solution, but in such case other management options also need to be considered, including habitat offsetting to compensate for losses, and full or partial barrier removal to reduce the scale and size of impoundments. Improvement of riparian ecotones may provide also a useful measure, which may create refugia for juvenile fish migrating downstream. We note that protection, restoration, and enhancement of fish habitats and riparian zones do not only improve biodiversity and ecosystem function. These measures also provide wider benefits by maintaining good water quality and other ecosystem services, such as recreation, fishing, and resilience to climate change. They also support wider management plans such as landscape and forest restoration.

Hence our results inform not only on specific strategies, such as locations where removal of barriers and improvement of riparian zones are desirable, but also provide a quantitative backbone for alternatives analysis. The *wRHp* can serve as a metric for comparing future management scenarios like it was demonstrated in a case study of the AMBER project (www.amber.international), where lowering of Włocławek Dam on the Vistula River to 30% of current height could be selected as most promising fish ecological restoration alternative^34^.

Our pan-European FCMacHT classification provides a consistent natural riverine habitat taxonomy for Europe, informed by robust data analysis of fish catch data (electrofishing surveys) and key environmental variables available at a continental scale. It allows differentiating barrier impacts according to the fish community structure expected at their geographical locations, documenting that not only barrier density matters in landscape-level assessment of damage. It is also important for prioritization of management actions. We envisage multiple uses of the FCMacHT typology and barrier impact assessment, from environmental and ecological studies, through river restoration planning, to water resources management and policy implementation (e.g., WFD or Water4All Partnership Program). Our barrier impact assessment is complementary to the guidelines for river-basin management plans^1,35,36^, which focus more on the passability of fish passage than habitat impact. It is our opinion that the determination of free-flowing river status (for which criteria are currently developed) should be tied to the FCMacHT characteristics, which define the habitat needs of expected fish community composition.

CHAMP is an innovative adaptation of physical habitat modeling methods. It introduces a concept of estimating in relative terms the magnitude and direction of alteration of free-flowing river habitats by human interventions. The model focuses on the change rather than on a detailed description of a newly modified state and goes straight to quantifying the proportion of habitat alteration. As such, the model concept is applicable globally and to many other aspects of impact assessment such as environmental flows, channelization, or water quality. Therefore, we expect and hope that our findings and approach will find broad applications that may go beyond rivers and river restoration.

## Methods

### Overview

This paper presents a riverine macrohabitat typology created in order to allow for barrier impact assessment on fish fauna in compliance with the EU WFD. It builds on the notion of a template habitat structure supporting an expected fish community composition. The typology can further be used to predict the level of “deviation” of freshwater communities from their predicted baseline. The habitat templates are predicted from broad physio-geographic and environmental features corresponding with fish catch data. The steps involved in the process together with input data sources are presented in Fig. 1 and are described in more detail further below.

Key assumptions and limitations regarding the assessment of the barrier impacts include the following:

1. Fish assemblages are considered good indicators of the environmental state of rivers^37^. Due to their high mobility and relatively long lifespan, fish use various habitats within river ecosystems, so they are sensitive to disturbances in-river morphology^38^. As the only riverine organisms that actively migrate long distances, fish are strongly affected by disturbance to the river continuum^39^.
2. While species composition varies between biogeographic regions, it is effective to assess fish assemblage responses to riverine habitat changes caused by barrier impacts on a continental scale using HUG.
3. The presented model of barrier impacts on fish habitats only considers the upstream changes to river hydromorphology in relation to European macrohabitat river types.
4. This is a landscape scale analysis investigating general patterns and does not account for locally specific and temporal habitat variability.

### Conceptual habitat alteration model for ponding

To determine the impact of barrier impoundments on habitat availability for HUG, we developed a conceptual habitat alteration model for ponding (CHAMP) using available scientific information. This was preceded by an extensive review of the literature with findings discussed in a technical workshop for fish biology experts involved in the AMBER project^40^. To create the model we used two types of data and a barrier typology:

### Fish data

The source of fishery data is the European Intercalibration database gathered between 2006 and 2011 under the auspices of the European Commission Joint Research Center (JRC) to support the implementation of the Water Framework Directive^41^. This database contains information on 4561 fish survey sites in 22 countries. The database consists of several tables describing basic physical parameters for each site, details of each fishing campaign, and catches obtained by electrofishing surveys. Data on anthropogenic pressures, including impounding, are available, thereby enabling distinction between undisturbed and disturbed sites. The Intercalibration database was used with the permission of JRC and data owners from 19 EU member states involved. A similar dataset for Poland, gathered during the auto-intercalibration process^42^, was used with the permission of the Chief Inspectorate for Environmental Protection, Poland.

The final database containing 5497 survey sites was subsequently filtered on the basis of pressure criterion in order to select anthropogenically undisturbed sites. Pressures were grouped into five categories: (i) connectivity, (ii) hydrological alteration, (iii) morphological alteration, iv) water quality, and v) navigation, recreation, and biological pressure from invasive species. Sites least impacted were considered references for the purpose of river ecosystem quality assessment. Of the 1315 sites (river reaches) meeting criteria for being least impacted, a subset of 1099 in 17 countries were identified considering data use limitations and data quality issues (i.e., precision of the site location referenced to the river network database, discussed below). The location of fishing sites is presented in Supplementary Figure 5.

### Broad-scale environmental influences on fish habitats

For the purpose of this study, we selected coarse-scale, hence unaffected by human disturbance, environmental characteristics of rivers, and their catchments to use as proxies for habitat structure. They include altitude and slope of river segment, stream order, catchment size, geology, and bioclimatic conditions. These features determine such habitat characteristics as flow velocity, riverbed width and depth, and longitudinal profile. In turn, catchment geology (i.e., the physical and chemical properties of rocks and soils) contributes to riverbed substrate material formation, relief of the catchment and drainage network density, shape of the river valley and riverbed cross-section, as well as the main physico-chemical character of surface waters. Finally, climatic variables, such as temperature, precipitation, and insolation intensities together with temporal patterns determine flow regime, physico-chemical properties of waters, vegetation, and phenological seasons. Attributes of land cover and water pollutants have not been selected as attributes, as they are the most sensitive to human-induced alteration.

The river network and its geometric characteristics were obtained from the River and Catchment Database derived from the Catchment Characterization Model (CCM, v2.1)^43^, which contains spatially attributed information on slope, altitude, Strahler stream order, and catchment area (Supplementary Figure 1, Supplementary Table 3).

Catchment geology was derived from the International Hydrogeological Map of Europe (IHME1500, v1.2) at a scale of 1:500,000^44–46^ and its structured lithological attributes of bedrock formations. Rock formations have been classified into calcareous and siliceous types based on Water Framework Directive recommendations for stream classification. Location of organic material was derived from the European Soil Database (ESDB, v2.0)^47^ based on soil type and the soil parent material attributes of soil mapping units. Details regarding classification of geological formations are shown in Supplementary Table 6. Geological type has been attributed to river reaches based on location of its geometric center. Where data for river reaches were missing, values were derived from the nearest river segments upstream that had recorded information.

Finally, climate-related factors were obtained from the Environmental Stratification of Europe^48^, where environmental zones have been delimited by modeling a set of environmental and climatic variables forming regions of homogeneous environmental conditions.

Geographic data available for the entire study area was used to assure best available data consistency among regions. Using pan-European datasets, however, often involves compromising on accuracy and precision. Even though we used the best available datasets, we are aware of several issues, which could affect our results but, which could not be resolved at the time of the analysis. In particular, river course precision and accuracy issues manifest themselves, especially in the low-relief terrain by incorrect alignment in vertical and longitudinal dimensions. This could, in some cases, lead to the point that a river misses its correct outflow, and in the worst case splits into two rivers flowing in two distinct directions, entailing wrong catchment attribution^49^. Low precision and accuracy of geology and soil data might have produced further inconsistencies with river network data. Some examples are depicted in Supplementary Figure 6. Calculation of percent error revealed high relative error for selected locations (Supplementary Table 5), which could not be corrected despite various quality checks (details in Supplementary Information). Spatial data preparation, handling, and analysis were all performed in ArcGIS Desktop 10.3^50^.

### River barrier types

Barriers are being constructed to serve multiple economic and societal goals. Their impact on river habitats depends to a greater or lesser extent on the magnitude and permanency of the impoundments they form. In our analysis, we consider six barrier types—dams, weirs, sluices, culverts, fords, and ramps—varying in function, size, and impoundment extent^22^. Three of them are usually constructed specifically to create impoundments (dams, weirs, and sluices). Dams are hydrotechnical structures designed to store water and create permanent impoundments^3^. They are the largest and least frequent barrier type in Europe^2^. Weirs are typically smaller and constructed to regulate flow conditions and upstream water levels. Sluices form a movable barrier (horizontal or vertical) aimed at controlling water levels and flow rates in rivers and streams. Culverts are designed to convey streams and small rivers through or under an obstruction, such as a road or dike. Fords create wadeable shallows for crossing a stream, while ramps (or bed sills) help stabilize the channel bed and reduce erosion^3^. The last three are the most common barriers in European rivers, yet their ponding effects are spatially and temporally restricted^2^.

### CHAMP model development

The model has been built in the following steps:

### Step 1 Defining HUGs

Fish surveys across a large region are affected by variability due to different species composition as well as sampling methodology, seasonality, and year of survey. This affects the accuracy of fish community prediction models. To account for this, predicted fish distributions can be fitted into generic distribution patterns such as those used in biocomplexity models^51^. The Target Fish Community approach^52^ uses a model of ranking relative abundance of species observed during a survey, which can, in turn, be used to calculate expected proportions according to a biocomplexity formula. Fish catch data were subject to this procedure to level out the influence of natural variability on fish catch data.

To further reduce regional variability caused by species composition, it has been proposed to group species into habitat-use guilds and estimate the structure of a guild assemblage rather than species. This approach was first used in Poland to determine environmental flow rules for the country^53,54^.

HUG were determined by modifying the fish guild classification created for the EFI+ Project^19^ and supplemented with other literature sources^55–57^. We began with the EFI+ Manual^20^, where each of the 302 species occurring in European rivers was ascribed to a guild: intolerant or tolerant species, benthic or rheophilic species, lithophilic or phytophilic species, and insectivorous or omnivorous species. Fish habitat preferences vary in terms of available food resources, substrate for spawning, flow characteristics, pelagic versus demersal or benthic lifestyles, and tolerance to physio-chemical (oxygen) and hydromorphological changes^55^. Therefore, grouping fish species into HUG was performed by combining particular guild characteristics (i.e., tolerance to environmental change, flow preference, and spawning and feeding behavior) to represent dominant habitat needs and a range of tolerance to environmental gradients (Supplementary Table 1). To capture temporally predominant habitat use, emphasis was given to foraging behavior as prevalent during the time of capture. Thus, while naming the guilds, we generalized the classification used for spawning preference description (e.g., phytophilic spawners) and applied it to adult life stages. Hence, phytophilic species are here considered to prefer vegetation cover during the rearing and growth life stage and lithophilic species as fish most commonly found over stony substrate. As a result eleven fish guilds were distinguished: intolerant highly rheophilic species, rheophilic benthic species preferring sandy-gravel bottom substrate, rheophilic water column species preferring sandy-gravel bottom substrate, limnophilic benthic species of moderate tolerance, limnophilic water column species of moderate tolerance, intolerant rheophilic benthic species preferring detritus or pelal bottom substrate, intolerant water column species, limnophilic lithophilic species of moderate tolerance, limnophilic phytophilic species of moderate tolerance, benthic species of moderate tolerance, and tolerant generalist species (Fig. 2). Fish species belonging to a particular HUG are provided in Supplementary Table 2 and a detailed description of the HUG together with their habitat preferences is provided in the Supplementary Information (Box 1).

### Step 2 Guild’s habitat suitability and its alteration by ponding

Using data from the available literature about habitat preferences for each HUG normally associated with free-flowing river fish habitats, we assessed how much these attributes are altered by impoundment created by a barrier type. To this end, we identified 21 key free-flowing riverine habitat criteria (habitat preferences), which are important for aquatic organisms in rivers and are likely to be modified by impounding. These are:

- flow velocity (high and low velocity)
- depth (deep and shallow areas)
- substrate (interstitial spaces, sandy or muddy bottom, and gravel)
- in-stream cover (woody debris or boulders)
- physico-chemical conditions (oxygenation, water temperature, and nutrient content)
- vegetation (rheophilic vegetation or mosses, macrophytes, canopy shading from banks, and overhanging vegetation)
- structure of banks (undercut banks)
- floodplain accessibility
- habitat continuity
- flow stability

For each HUG, these criteria were assessed for their importance to fish presence and abundance according to information found in the literature. Subsequently, for each habitat criterion, a score was assigned to obtain a habitat preference index (*HP*) in the following scale:

0 = not important

0.5 = moderately important

1 = very important

HP reflects the importance of habitat criteria to fish presence, but not how these criteria change in response to the presence of the impoundment. In turn, habitat alteration in terms of a relative change was assessed for each barrier type from major reduction to major increase, using five categories ranging in value from 0 to 2, to obtain a Habitat Alteration (*HA*) index:

0.0 = major reduction

0.5 = small reduction

1.0 = no change

1.5 = small increase

2.0 = major increase

The selection of the value range was guided by the fact that impoundment can have a negative impact on some fish groups through reduction of area with suitable attributes (e.g. less area with high velocities), hence the loss of lotic habitats, while other guilds (i.e., generalists and limnophilic phytophilic species) can benefit from changes caused by the presence of a barrier. Accordingly, the HUG’s response to a barrier can be either negative or positive because of habitat becoming more or less suitable.

Combining the two indices (*HP* and *HA*) produces the Altered Habitat Suitability index (*AHS*) for HUGs affected by pressures from different barrier types (see Supplementary Data 1). It can be interpreted as a suitability score of altered habitat of the guild. When compared to unaltered habitat it indicates a proportion of remaining unaltered habitat area of a guild at a barrier type. For example, for rheophilic species high water velocity was determined to be a critically important habitat attribute (${HP}=1$). Since the presence of dams causes impoundment of water and decreases flow velocity, this results in a major reduction of habitat area with this particular habitat feature (*HA* = 0) and in turn, high suitability reduction (*AHS* = 1 × 0 = 0). Similarly, macrophytes are moderately important for limnophilic benthic species of moderate tolerance (*HP* = 0,5). While dams increase macrophytes abundance to a large extent (*HA* = 2), the *AHS* for this attribute is 1, indicating that the habitat suitability dependent on this attribute will not substantially change under impoundment. For phytophylic species (*AHS* = 1), the suitable habitat area would double. Hence, the *AHS* value above 1 would indicate an increase of suitable habitat area due to an attribute change. In case a habitat feature is not important for a specific guild the *AHS* value would equal zero, thus not affecting the overall suitable habitat area. The sum of ${AHS}$ scores for HUG indicates a composite habitat suitability alteration due to the construction of a particular barrier type. To derive a measure of remaining riverine habitat proportion (*RHp*) for each HUG, *Composite AHS* values were normalized by sum of HP indices for the guild i.e. unaltered habitat suitability. Therefore *RHp* is calculated following the formula (1):

1 $${{RHp}}_{i,b}=\frac{{\sum }_{1}^{{{{{{\rm{j}}}}}}}\left({{{{{{\rm{HP}}}}}}}_{i,\, j}\times {{{{{{\rm{AH}}}}}}}_{b,\, j}\right)}{{\sum }_{1}^{{{{{{\rm{j}}}}}}}{{{{{{\rm{HP}}}}}}}_{i,\, j}}$$

where:

*RHp*_*i,b*_—remaining riverine habitat proportion (%) for barrier *b* and guild *i*

*HP*_*i,j*_—Habitat Preference index of habitat attribute *j* for guild *i*

*AH*_*b,j*_—Habitat Alteration index for habitat attribute *j* for barrier *b*

### Step 3 Clustering Fish Community MacroHabitats

A two-step cluster and discriminant analysis were applied to establish the relationship between catchment environmental settings and expected fish macrohabitats (using observed fish community structure as a proxy). In the first step, non-hierarchical cluster analysis was conducted using the Intercalibration data from 1099 river sites classified as Non-Disturbed Sites in the database. Cluster analysis was applied to two datasets sequentially. The first dataset consisted of physical attributes of the sampling sites obtained from the broad-scale environmental attributes described previously. This was clustered into samples with similar habitat characteristics (Supplementary Figure 2). After closer examination, it was determined that slope and altitude reduced model performance and had no influence on the results. Therefore, these two variables were excluded from the final model calculations and used only for better cluster characteristics description. Cluster groupings were then added as an additional variable to the biological data on HUG proportions at each site (obtained from the Intercalibration dataset of undisturbed sites) to produce a mixed dataset of guilds/physical clusters (Supplementary Figure 3). This second dataset was then clustered, resulting in one of 15 fish community macrohabitat types (FCMacHT) classes assigned to each sampling site. The HUG proportions expected to occur within a river reach of a specific habitat composition were determined using a Target Fish Community method for each FCMacHT. For each river type, 10 Non-Disturbed Sites (*n)* were selected at random, from which the sum of guild proportions was calculated and ranked, and the reciprocal rank calculated. The number of sample sites is based on the meta-analysis of publicly available Target Fish Community reports from rivers (*n* = 22) in New Hampshire (https://www.des.nh.gov/resource-center/publications?keys=tfcrpt). In these studies, *n* was defined using Multivariate Pseudo Standard Error (MultSE^58^) and only rarely exceeded *n* = 10. Expected guild proportions were then obtained by dividing each guild reciprocal rank by the sum of the reciprocal ranks and plotted as pie charts for each FCMacHT (Fig. 2).

A distance matrix was created by standardizing the data using Gower and Manhattan similarity distances for the physical and mixed datasets, respectively^59,60^. The number of clusters was determined with the help of scree and silhouette plots. A partitioning around medoids (PAM) clustering model was applied^61^. Cluster plots and silhouette plots, as well as box plots, were created for each variable. Subsequently, data discrimination with analysis of similarities (ANOSIM) was performed to verify model performance (Supplementary Figure 2, Supplementary Figure 3).

By clustering these physical attributes together with pan-European fish survey data grouped into HUG, the fish macrohabitat classification model for Europe was created to estimate fish community structure expected under natural reference conditions. The analysis produced a robust and accurate model (ANOSIM *R* = 0.98, *p* < 0.001) of 15 FCMacHT, which are uniquely characterized by different proportions of HUG.

### Step 4 Geographic distribution of Fish Community Macrohabitat Types in Europe—FCMacHT

Finally, the relationship between macrohabitat type and broad-scale environmental attributes was analyzed to define a riverine macrohabitat typology for the entire European river network. To this end, the FCMacHT class was added to the physical attributes of each undisturbed site as a grouping variable to perform CART analysis^62^ for determining how FCMacHT is distributed along the gradients for physical descriptors (Kappa = 0.86, Supplementary Figure 4). A complexity parameter plot was used to determine an acceptable relative error for pruning the decision tree. Following this, the decision tree was applied to all water bodies of Europe with specified environmental attributes to determine their FCMaCHT class (Supplementary Information, Box 2). A summary of basic statistics of the Non-Disturbed Site dataset in comparison with the extrapolated dataset is shown in Supplementary Table 3. Statistics for each FCMacHT class are presented in Supplementary Table 4.

### Step 5 Assess the regional sensitivity of fish communities to habitat change

The summed *HP* value for each guild reflects the suitability of river habitat attributes for a given fish guild, forming Ponding Sensitivity Index (*PSI*, compare Supplementary Data 1 for details). This value is highest for specialized river guilds and lowest for generalists. The PSI served as standardization factor for calculating *RHp* values (Formula 1). To evaluate regional sensitivity of fish communities to habitat change, we weighted *PSI* with the expected proportion of each guild for a given FCMacHT and divided it by the maximum suitability value of 21, which assumes all attributes are very important (Formula 2). These relative sensitivity levels were then split into three classes: lowest, moderate, and highest with values <45%, between 45% and 55%, and >55%, respectively (Fig. 4, Supplementary Data 1).

2 $${FCMacHT}\,{sensitivity}=\frac{\mathop{\sum }\nolimits_{1}^{i}({{PSI}}_{i}\times {{GP}}_{i})}{21}\times 100$$

where:

*PSI*_*i*_—Ponding Sensitivity Index for guild *i*

*GP*_*i*_—Habitat Use Guild *i* proportion in a FCMacHT

### Step 6 Estimate barrier-specific fish community habitat alteration at impoundments

Also guild specific *RHp* values were further weighted by HUGs proportions at a given location and summed for all guilds to determine an overall weighted Riverine Habitat proportion (${wRHp}$) for a given FCMacHT using the following formula (3):

3 $${wRHp}={\sum }_{1}^{i}({{GP}}_{i}\times {{RHp}}_{i,b})$$

where:

*wRHp*—weighted Riverine Habitat proportion (%) in a FCMacHT for barrier *b*

*GP*_*i*_—Habitat Use Guild proportion of guild *i* (%)

*RHp*_*i,b*_—remaining riverine habitat proportion (%) for barrier *b* and guild *i*.

This formula was used to estimate the impact of each barrier type on habitat suitability and availability for the expected fish community of each FCMacHT river type, expressed as the percentage of the remaining suitable habitat for fish communities.

Values for ${wRHp}$ range from 0 to 200%, with 0% indicating no suitable habitat due to full alteration of all habitat attributes important to each fish guild, 100% indicating no barrier impact, and 200% corresponding to a theoretical major increase of suitable area (for generalist species for example). None of the obtained values reached the 200%, and only fords and ramps were close to no impact value in most of the locations (Table 1).

### Step 7. Estimating free-flowing river habitat alteration of European rivers

The AMBER Barrier Atlas^22^ was used to identify existing barriers in Europe. The Atlas contains all barriers included in public inventories, be it state-owned, international, or regional. The barriers with known barrier type were attributed with the FCMacHT of the catchment they belong to, and with ${wRHp}$ assigned on the basis of Table 1. Values of ${wRHp}$ for each barrier were plotted spatially on the map and the median value was used to color code catchments (Fig. 6).

Using the AMBER Barrier Atlas, we were also able to determine known barriers’ height and estimate the extent of the backwater effect of dams, weirs, and sluices using pan-European river network and gradient data^43^. For the 92,167 (37%) dams, weirs, and sluices recorded in the AMBER Barrier Atlas^2^ we obtained height measurements and combined them with river slope values available from the river network. Missing data estimate was accounted for.

Barrier height was taken as the difference between the bottom of the riverbed and the lowest point on the crest of the barrier. The impoundment length was, in turn, estimated by dividing barrier height by slope. Total impoundment length for each barrier type was then corrected for barrier-specific reporting errors published in Belletti et al.^2^, as well as missing barrier height data within the AMBER Barrier Atlas (Table 2). Total river habitat alteration in Europe (*HAB*_*alt*_), measured in km, was calculated with the formula:

4 $${{HAB}}_{{alt}}={\sum }_{1}^{T}\left(\frac{{\sum }_{1}^{b}\left(\frac{h}{s}\right)}{\left(1-{{Err}}_{b}\right)\times {p}_{b}}-\,\frac{{\sum }_{1}^{b}\left(\frac{h}{s}\times {{wRHp}}_{b}\right)}{\left(1-{{Err}}_{b}\right)\times {p}_{b}}\right)$$

where:

*h*—barrier height (m)

*s*—river slope (‰)

*Τ*—number of barrier types, indexed by $b$

*wRHp*_*b*_—weighted Riverine Habitat proportion (%) for barrier type $b$

*p*_*b*_—the proportion of barriers (%) of type $b$ with height information

*Err*_*b*_—reporting error for barrier type *b* based on modeled barrier density^2^.

The simplified formula used for estimating the impoundment length ($h/s$) of individual barriers was compared with two similar formulas used in the field of hydraulic engineering (Supplementary Table 7) and is discussed in the Supplementary Information.

### Reporting summary

Further information on research design is available in the Nature Portfolio Reporting Summary linked to this article.

### Supplementary information

Supplementary information

Description of Additional Supplementary Files

Supplementary Data 1

Reporting Summary

## Data Availability

Fish catch data and location of non-disturbed sites were obtained from EU fish-based River Ecological Quality assessment referenced here as intercalibration dataset (IC). While there is a central dataset maintained at the Joint Research Center, data copyrights belong to individual countries or (in case of Germany) individual administrative units. We obtained written permission to reuse the data from individual entities with no further right to republish the data. Access to these data requires a written request to JRC, contact person Wouter van de Bund (Wouter.VAN-DE-BUND@ec.europa.eu). More information at https://www.nffa.eu/about/consortium/site/?id=43. The broad environmental characteristics of the river sites and non-disturbed sites were acquired from the River and Catchment Database derived from the Catchment Characterization Model (CCM2.1)^43^, the International Hydrogeological Map of Europe (IHME1500, v1.2) at a scale of 1:500,000^44^, European Soil Database (ESDB, v2.0)^47,63^, the Environmental Stratification of Europe^48^. The variables derived from these sources and used for the analysis are made available under a CC-BY-4.0 license here: 10.6084/m9.figshare.22730897^64^. Barrier data (Fig. 6) as well as the underlying data for Table 2 come from the AMBER Barrier Atlas and are freely available at https://amber.international/european-barrier-atlas/ under a CC-BY-4.0 license and here: 10.6084/m9.figshare.12629051.v5^65^. Data used for generation of Fish Community Macrohabitat Types map (Fig. 3), fish sensitivity to impounding map (Fig. 4), estimated barrier habitat impact across European rivers with respect to barrier type (Fig. 5), projected barrier-level impounding impacts in European catchments (Fig. 6), and river segment’s and catchment’s characteristics used as proxies for delimitation of macrohabitat types (FCMacHTs) in European rivers (Supplementary Fig. 1) are made available under a CC-BY-4.0 license here: 10.6084/m9.figshare.22730897^64^.

## Code availability

The R code used to perform cluster and discriminant analysis of fish guilds, catchments environmental settings, and expected fish macrohabitats is available under a GNU General Public License v 3.0 here 10.5281/ZENODO.8004302^66^. ArcGIS Toolbox used to automate the calculation of Fish Community Macrohabitat Types (FCMacHT) over non-disturbed sites and European river network based on the Classification and Regression Trees model is available under a GNU General Public License v 3.0 here: 10.5281/zenodo.8171043^67^. The calculation of the remaining attributes was made manually in ArcGIS Desktop 10.3.1 using ad hoc procedures and is not deposited in a repository.
