# Supplementary material for: Over 200,000 kilometers of free-flowing river habitat in Europe is altered due to impoundments
Source: Nat Commun. 2023 Oct 9;14:6289. doi: 10.1038/s41467-023-40922-6 (PMC10562483; doi:10.1038/s41467-023-40922-6)
Supplement: Supplementary file 1 — Supplementary information [file 41467_2023_40922_MOESM1_ESM.pdf]

Over 200,000 kilometers of free-flowing river habitat in Europe is altered due to impoundments. Supplementary information

Piotr Parasiewicz[1] , Kamila Belka[1,2], Małgorzata Łapińska[3,2], Karol Ławniczak[2], Paweł Prus[1], Mikołaj Adamczyk[1], Paweł Buras[1], Jacek Szlakowski[1], Zbigniew Kaczkowski[2,3], Kinga Krause[2], Joanna O'Keeffe[1] , Katarzyna Suska[1], Janusz Ligięza[1], Andreas Melcher[4], Jesse O'Hanley[5], Kim Birnie-Gauvin[6], Kim Aarestrup[6], Peter E. Jones[7], Joshua Jones[7], Carlos Garcia De Leaniz[7], Jeroen S. Tummers[8,9], Sofia Consuegra[7], Paul Kemp[10], Hannah Schwedhelm[11], Zbigniew Popek[12], Gilles Segura[13], Sergio Vallesi[8,14], Maciej Zalewski[2], Wiesław Wiśniewolski[1]

[1] National Inland Fisheries Research Institute, Poland
email: p.parasiewicz@infish.com.pl (for author 1), k.belka@infish.com.pl

[2] European Regional Centre for Ecohydrology of the Polish Academy of Sciences, Poland
[3] University of Lodz, Łódź, Poland
[4] University of Natural Resources and Life Sciences, Vienna, Austria
[5] University of Kent, Canterbury, UK
[6] Technical University of Denmark, Silkeborg, Denmark
[7] Swansea University, Swansea, UK
[8] Durham University, Durham, UK
[9] RAVON, Nijmegen, The Netherlands
[10] University of Southampton, Southampton, UK
[11] Technical University of Munich, Germany
[12] Warsaw University of Life Sciences, Poland
[13] IS Environnement, Saint-Jean, France
[14] Hydronexus, Bologna, Italy

1. Habitat use guilds

Fish assemblages are considered good indicators of a river's environmental condition as well as riverine habitat suitability and availability [1–3]. There are several advantages of using fish as indicator organisms[4,5] and can be regarded as an "umbrella group" for other aquatic organisms. Relatively long-lived and present in almost all lotic ecosystems, fish typically reflect the cumulative effects of long-term anthropogenic stressors[6–8]. Being at the high end of the trophic pyramid, fish are regulated by and respond to changes of lower tropic levels, including benthic invertebrates and zooplankton, which is a food resource for non-predatory species[9]. Some fish species also feed on algae and higher aquatic plants, but macrophytes are mostly important as habitat structural elements and spawning substrate. Due to their high mobility and use of various habitats within river ecosystems, fish are also particularly sensitive to disturbances in river morphology[10]. As the only riverine organisms that actively migrate long distances, fish are also strongly affected by river continuum disturbances[11].

Fish species richness in European inland waters is limited (381 species total) compared to other regions around the globe and in most cases species are easy to identify taxonomically at the species level[12]. However, species composition varies greatly between biogeographic regions. Therefore, to assess fish assemblage responses to riverine habitat changes caused by human impacts on a continental scale, definition of Habitat Use Guilds is a generally more useful approach (see Supplementary Table 1, Box 1)[13]. Fish species belonging to a particular Habitat Use Guild are provided in Supplementary Table 2.

Supplementary Table 1. **Habitat Use Guilds characteristics**. They were distinguished on the basis of Melcher's guild classification for the Expected Fish Communities development.

No.	Habitat Use Guild	Functional guilds *							
		**Intolerant**	**Tolerant**	**Benthic**	**Rheophilic**	**Lithophilic**	**Phytophilic**	**Omnivorous**	**No. Species**
1.	Intolerant highly rheophilic species	1	0	x	1	1	0	0	14
2.	Rheophilic benthic species preferring sandy-gravel bottom substrate	x	x	1	1	0	x	0	56
3.	Rheophilic water column species preferring sandy-gravel bottom substrate	x	x	0	1	1	0	x	24
4.	Limnophilic benthic species of moderate tolerance	x	0	1	0	0	0	0	28
5.	Limnophilic water column species of moderate tolerance	x	0	0	0	x	0	x	41
6.	Intolerant rheophilic benthic species preferring detritus or pelal bottom substrate	1	0	1	1	1	0	0	10
7.	Intolerant water column species	1	0	0	x	x	x	x	17
8.	Limnophilic lithophilic species of moderate tolerance	0	0	0	0	1	0	x	6
9.	Limnophilic phytophilic species of moderate tolerance	x	0	x	0	0	1	x	26
10.	Benthic species of moderate tolerance	x	0	1	x	x	x	x	16
11.	Tolerant generalist species	0	1	x	x	x	x	x	10

* 1 – species belong to a guild, 0 – species do not belong to a guild, x – guild not taken into account

Box 1. **Description of Habitat Use Guilds within the riverine macrohabitat typology.** Each guild has specific habitat requirements to thrive and survive.

1) *Intolerant highly rheophilic species* guild includes species that are pollution intolerant, rheophilic and/or lithophilic. This guild requires high water velocity, gravel bottom substrate and interstitial space, low water trophy and temperature, and high oxygen concentrations. In addition, included species require shelter availability and longitudinal connectivity.

2) *Rheophilic benthic species preferring sandy-gravel bottom substrate* guild consists of only rheophilic species foraging at the river bottom, excluding all lithophilic and omnivorous species regardless of other preferences. This guild shows similar preferences to habitat attributes as the one above. In addition, these species require increased depth, the presence of rheophilic macrophytes and mosses, as well as habitat stability.

3) *Rheophilic water column species preferring sandy-gravel bottom substrate* guild consists of species inhabiting the open water column of fast-flowing rivers with available sandy-gravel bottom. This guild shows complementary preferences to those described above, but benefits from more depth and slightly slower velocity. In addition, this guild has relaxed requirements regarding water quality, however, these species require gravel areas and interstitial spaces for spawning, as well as cover and habitat stability.

4) *Limnophilic benthic species of moderate tolerance* guild consist of fish that prefer living in slow moving, still or stagnant water, excluding rheophilic, litophilic, phytophilic or omnivorous species. This guild is associated with lentic habitats with low water velocity, high depth, and soft bottom sediment. It has low water quality and habitat continuity requirements.

5) *Limnophilic water column species of moderate tolerance* guild includes species that are not intolerant, not benthic, not rheophilic, and not phytophilic. This group is associated with soft bottom substrates, macrophytes, and floodplain water bodies. It tolerates habitat fragmentation and instability.

6) *Intolerant rheophilic benthic species preferring detritus or pelal bottom substrate* guild is composed of lamprey species, with specific biology and habitat requirements. Larvae are detritivorous and inhabit shallow areas. Some of them are also long-migratory species, with a marine, parasitic adult phase. Feeding habitats in rivers are specific to the detritivorous larval stage. Adults highly differ in feeding strategy with parasitic forms (usually marine) or those that do not feed at all (resorbed digestive track) during a short adult stage. This guild requires moderate to high water velocity accompanied by shallow

margins or backwaters with more lentic conditions. It needs a muddy or detritus substrate and good water quality and oxygen conditions. This group is dependent on natural hydromorphologic conditions and vulnerable to habitat modifications, especially changes in water depth and substrate composition. It is also sensitive to habitat fragmentation.

7) *Intolerant water column species* guild consists of pollution intolerant species and those that are not benthic. This guild needs good water quality, low temperature, cover, and well-preserved longitudinal connectivity. It also needs moderate water velocity and coarse sediment and is sensitive to changes in composition towards sand and mud.

8) *Limnophilic lithophilic species of moderate tolerance* guild consists of lithophilic species that are neither highly tolerant nor intolerant. They are also neither benthic nor rheophilic nor phytophilic. This guild is associated with coarse bottom substrate with affinity for low water velocity and high water depth. It requires some cover, especially woody debris. This guild is also very sensitive to river fragmentation and habitat instability.

9) *Limnophilic phytophilic species of moderate tolerance* guild includes phytophilic species which are neither tolerant, nor rheophilic, nor lithophilic. This guild prefers lentic habitats with aquatic vegetation, low water velocity, greater depth and soft bottom sediment. It tolerates increased water trophy, higher temperatures and lower oxygen content. The guild is less sensitive to disruption of longitudinal river continuity, but it is strongly dependent on floodplain waterbodies, so lateral connectivity is essential.

10) *Benthic species of moderate tolerance* guild consist of benthic species that are not tolerant. This guild is associated with medium water velocity and depth as well as bottom habitats. It prefers coarse bottom sediment and is strongly dependent on shelter. It has moderate requirements for water quality and habitat continuity, but requires stable habitat conditions.

11) *Tolerant generalist species* guild contains only ubiquitous species that are highly tolerant to habitat modification. This guild has no clear habitat preferences. It tolerates lentic and moderately lotic habitats, preferring higher depth and the presence of aquatic vegetation. It is not affected by the change of substrate composition towards soft bottom sediments. This guild tolerates high water pollution, higher temperatures and low oxygen concentration. It is not sensitive to disruption of longitudinal and lateral river continuity or to unstable habitat conditions.

Supplementary Table 2. **Fish species assigned to Habitat Use Guilds.**

1 **Intolerant highly rheophilic species** (n=14)			
Barbus caninus	*Parachondrostoma miegii*	*Salmo obtusirostris*	*Telestess souffia*
Cobitis calderoni	*Romanichthys valsanicola*	*Salmo salar*	*Thymallus thymallus*
Cottus poecilopus		*Salmo trutta fario*	*Zingel streber*
Hucho hucho		*Salmo trutta trutta*	*Zingel zingel*
2 **Rheophilic benthic species preferring sandy-gravel bottom substrate** (n=56)			
Acipenser gueldenstaedtii	*Barbus peloponnesius*	*Huso huso*	*Romanogobio albipinnatus*
Acipenser naccarii	*Barbus plebejus*	*Luciobarbus steindachneri*	*Romanogobio banarescui*
Acipenser nudiventris	*Barbus tyberinus*	*Oxynoemacheilus bureschi*	*Romanogobio benacensis*
Acipenser oxyrinchus	*Chondrostoma knerii*	*Oxynoemacheilus pindus*	*Romanogobio elimeius*
Acipenser ruthenus	*Chondrostoma nasus*	*Pachychilon pictum*	*Romanogobio kesslerii*
Acipenser stellatus	*Chondrostoma soetta*	*Parachondrostoma arrigonis*	*Romanogobio uranoscopus*
Acipenser sturio	*Chondrostoma vardarense*	*Parachondrostoma toxostoma*	*Rutilus pigus*
Ballerus sapa	*Cobitis elongata*	*Protochondrostoma genei*	*Sabanejewia aurata*
Barbatula barbatula	*Cobitis vardarensis*	*Pseudochondrostoma polylepis*	*Sabanejewia balcanica*
Barbus barbus	*Cobitis vettonica*	*Pseudochondrostoma willkommii*	*Sabanejewia romanica*
Barbus cyclolepis	*Cottus gobio*		*Vimba melanops*
Barbus euboicus	*Cottus koshewnikovi*		*Vimba vimba*
Barbus guiraonis	*Cottus petiti*		*Zingel asper*
Barbus haasi	*Gobio gobio*		*Zingel balcanicus*
Barbus macedonicus	*Gymnocephalus baloni*		
Barbus meridionalis	*Gymnocephalus schraetser*		

3 **Rheophilic water column species preferring sandy-gravel bottom substrate** (n =24)			
Achondrostoma arcasii	*Iberocypris palaciosi*	*Leuciscus pleurobipunctatus*	*Squalius illyricus*
Achondrostoma oligolepis	*Leuciscus aspius*	*Phoxinus phoxinus*	*Squalius keadicus*
Alburnoides bipunctatus	*Leuciscus burdigalensis*	*Rutilus frisii*	*Squalius lucumonis*
Alburnus albidus	*Leuciscus idus*	*Rutilus rubilio*	*Squalius torgalensis*
Anaecypris hispanica	*Leuciscus leuciscus*	*Squalius aradensis*	*Telestes fontinalis*
	Leuciscus muticellus	*Squalius cephalus*	*Telestes montenigrinus*
			Telestes polylepis
4 **Limnophilic benthic species of moderate tolerance** (n=28)			
Aulopyge huegelii	*Cobitis ohridana*	*Gymnocephalus cernua*	*Knipowitschia panizzae*
Cobitis arachthosensis	*Cobitis punctilineata*	*Iberochondrostoma lemmingii*	*Knipowitschia thessala*
Cobitis bilineata	*Cobitis stephanidisi*	*Knipowitschia caucasica*	*Luciobarbus albanicus*
Cobitis dalmatina	*Cobitis strumicae*	*Knipowitschia goerneri*	*Platichthys flesus*
Cobitis elongatoides	*Cobitis taenia*	*Knipowitschia milleri*	*Sabanejewia bulgarica*
Cobitis hellenica	*Cobitis tanaitica*		*Sabanejewia larvata*
Cobitis meridionalis	*Cobitis trichonica*		*Sander volgensis*
Cobitis narentana	*Cobitis zanandreai*		
5 **Limnophilic water column species of moderate tolerance** (n=41)			
Alburnus belvica	*Dicentrarchus labrax*	*Pelasgus minutus*	*Rutilus ylikiensis*
Atherina boyeri	*Iberochondrostoma lusitanicum*	*Pelasgus prespensis*	*Sander lucioperca*
Atherina hepsetus	*Ladigesocypris ghigii*	*Pelasgus stymphalicus*	*Squalius microlepis*
Atherina presbyter	*Liza aurata*	*Pelecus cultratus*	*Squalius svallize*
Ballerus ballerus	*Liza ramada*	*Phoxinellus alepidotus*	*Squalius zrmanjae*
Chelon labrosus	*Liza saliens*	*Rutilus basak*	*Syngnathus abaster*
Chondrostoma phoxinus	*Mugil cephalus*	*Rutilus heckelii*	*Telestes beoticus*
Chondrostoma prespense	*Pachychilon macedonicum*	*Rutilus karamani*	*Telestess croaticus*
Delminichthys adspersus	*Pelasgus epiroticus*	*Rutilus meidingeri*	*Telestes metohiensis*
Delminichthys ghetaldii		*Rutilus ohridanus*	*Telestes turskyi*
		Rutilus prespensis	*Telestes ukliva*
6 **Intolerant rheophilic benthic species preferring detritus or pelal bottom substrate** (n=10)			
Eudontomyzon danfordi	*Eudontomyzon stankokaramani*	*Lampetra fluviatilis*	*Lethenteron camtschaticum*
Eudontomyzon hellenicus	*Eudontomyzon vladykovi*	*Lampetra planeri*	*Petromyzon marinus*
Eudontomyzon mariae		*Lampetra zanandreai*	
7 **Intolerant water column species** (n=17)			
Alosa alosa	*Alosa maeotica*	*Coregonus autumnalis*	*Coregonus pidschian*
Alosa fallax	*Alosa tanaica*	*Coregonus lavaretus*	*Coregonus trybomi*
Alosa immaculata	*Alosa vistonica*	*Coregonus muscun*	*Osmerus eperlanus*
Alosa killarnensis	*Coregonus albula*	*Coregonus oxyrinchus*	*Salmo trutta lacustris*
			Salvelinus alpinus
8 **Limnophilic lithophilic species of moderate tolerance** (n=6)			
Alburnus chalcoides	*Coregonus peled*	*Squalius carolitertii*	
Alosa macedonica	*Petroleuciscus borysthenicus*	*Squalius pyrenaicus*	
9 **Limnophilic phytophilic species of moderate tolerance** (n=26)			
Aphanius fasciatus	*Leucaspius delineatus*	*Scardinius erythrophthalmus*	*Tinca tinca*
Aphanius iberus	*Misgurnus fossilis*	*Scardinius graecus*	*Tropidophoxinellus hellenicus*
Carassius carassius	*Pungitius hellenicus*	*Scardinius racovitzai*	*Tropidophoxinellus spartiaticus*
Economidichthys pygmaeus	*Pungitius platygaster*	*Scardinius scardafa*	*Umbra krameria*
Economidichthys trichonis	*Rhodeus amarus*	*Silurus aristotelis*	*Valencia hispanica*
Esox lucius	*Rutilus aula*	*Silurus glanis*	*Valencia letourneuxi*
	Scardinius acarnanicus	*Squalius alburnoides*	

10 **Benthic species of moderate tolerance** (n=16)			
Anguilla anguilla	*Luciobarbus comizo*	*Luciobarbus sclateri*	*Proterorhinus semilunaris*
Babka gymnotrachelus	*Luciobarbus graecus*	*Neogobius fluviatilis*	*Salaria fluviatilis*
Barbus prespensis	*Luciobarbus graellsii*	*Neogobius kessleri*	*Triglopsis quadricornis*
Lota lota	*Luciobarbus microcephalus*	*Neogobius melanostomus*	
Luciobarbus bocagei			
11 **Tolerant generalist species** (n=10)			
Abramis brama	*Carassius gibelio*	*Gasterosteus aculeatus*	*Pungitius pungitius*
Alburnus alburnus	*Cobitis paludica*	*Perca fluviatilis*	*Rutilus rutilus*
Blicca bjoerkna	*Cyprinus carpio*		

2. Fish Community Macrohabitat Types (FCMacHTs)

Freshwater ecosystems are regulated by multiple abiotic and biotic factors that depend directly on available habitat. Habitats, meanwhile, are formed by a hierarchy of processes (ecosystem drivers) that vary from local to the coarse scale[14–16]. Regional scale drivers such as climate and geographical barriers introduce variability within freshwater ecosystems that is indirectly related to local physical habitat conditions. Accordingly, different species may utilize similar habitat niches in different geographical locations. In view of this, our aim was to characterize the habitat makeup that would be capable of supporting a freshwater community template[17,18] as opposed to a particular set of species. This led us to propose a riverine macrohabitat typology for European rivers, referred to here as Fish Community Macrohabitat Types (FCMacHTs), to describe the expected habitat structure associated with a given Habitat Use Guild. Our FCMacHT classification system can further be used to evaluate anthropogenic alteration at continental scales by comparing with observed habitat structure.

In order to develop a macrohabitat typology, it is necessary to define a set of reference conditions. This usually involves estimation of the level of human induced alteration, with the assumption that low pressure defines reference sites and fauna sampled at low pressure sites represent reference conditions for the site[19,20]. Within the European Intercalibration exercise led by the ECOSTAT Working Group this approach has been used to develop EFI+ (European Fish Index Extension), which quantifies the departure of fish fauna from reference conditions.

An alternative approach has been developed recently that aims to predict the distribution of macrohabitats necessary for supporting a specific expected fish community structure within macroscale physiographic settings. These selected physical attributes that are deemed to be insensitive to anthropogenic pressures (e.g. watershed area, gradient, elevation). Deviation of local communities from such expected assemblage can be easily measured and linked to habitat alterations.

A prime example of such an approach is the one developed to predict expected proportions of fish species and diversity index for stream reaches in the Laurentian Great Lakes basin[21–25]. Using machine learning algorithms (neural networks and decision trees), the methodology was capable of explaining between 49 and 99% of calibration data variability. Neighboring stream reaches with similar predicted macrohabitats and communities were subsequently aggregated into a number of Fisheries Management and Conservation units via clustering. Lek and co-authors[26] present a similar approach for modelling aquatic fauna communities in France based on environmental characteristics. They argue that "the structure and diversity of aquatic communities in running waters are primarily dependent on a complex of physical, chemical and biotic factors"[26]. Their approach aims at predicting natural fish community structure inferred from a range of environmental variables, however, the variables considered are generally too fine scale and thus easily susceptible to human induced alteration.

Suitability of habitat for any particular biota or community of fish species is strongly dependent on the hydromorphological features of a river course. Water depth, flow velocity, composition of bottom substrate and macrophytes, presence of floodplain, dissolved oxygen, bank morphology, river corridor characteristics, longitudinal continuity, and habitat stability are all influenced by the interplay of large scale topographic, geologic, and climatic features and processes[27,28]. Local climate regulates the hydrological regime, which together with topography and geology directly affects water depth and velocity[29–31]. Depending on geology, these hydraulic parameters determine the type of bottom

substrate and shape of the river[32]. The amount of water in the river is also controlled by the size of the river catchment. Local climate, on the other hand, depends mainly on geographic location and altitude. Climatic variability influences the variability of the hydrological regime.

Given all this, we chose to follow the McKenna et al.[28] approach and select broad scale hydromorphologic and landscape level attributes such as stream order, elevation, watershed area, geology, and climate as predictors of habitat distribution patterns (Supplementary Figure 1). After building and validating the model, we investigated more closely the underlying physical patterns such as abundance of specific substrate types, geographic location, and climate zones in order to better describe core characteristics associated with individual FCMacHTs (see Box 2).

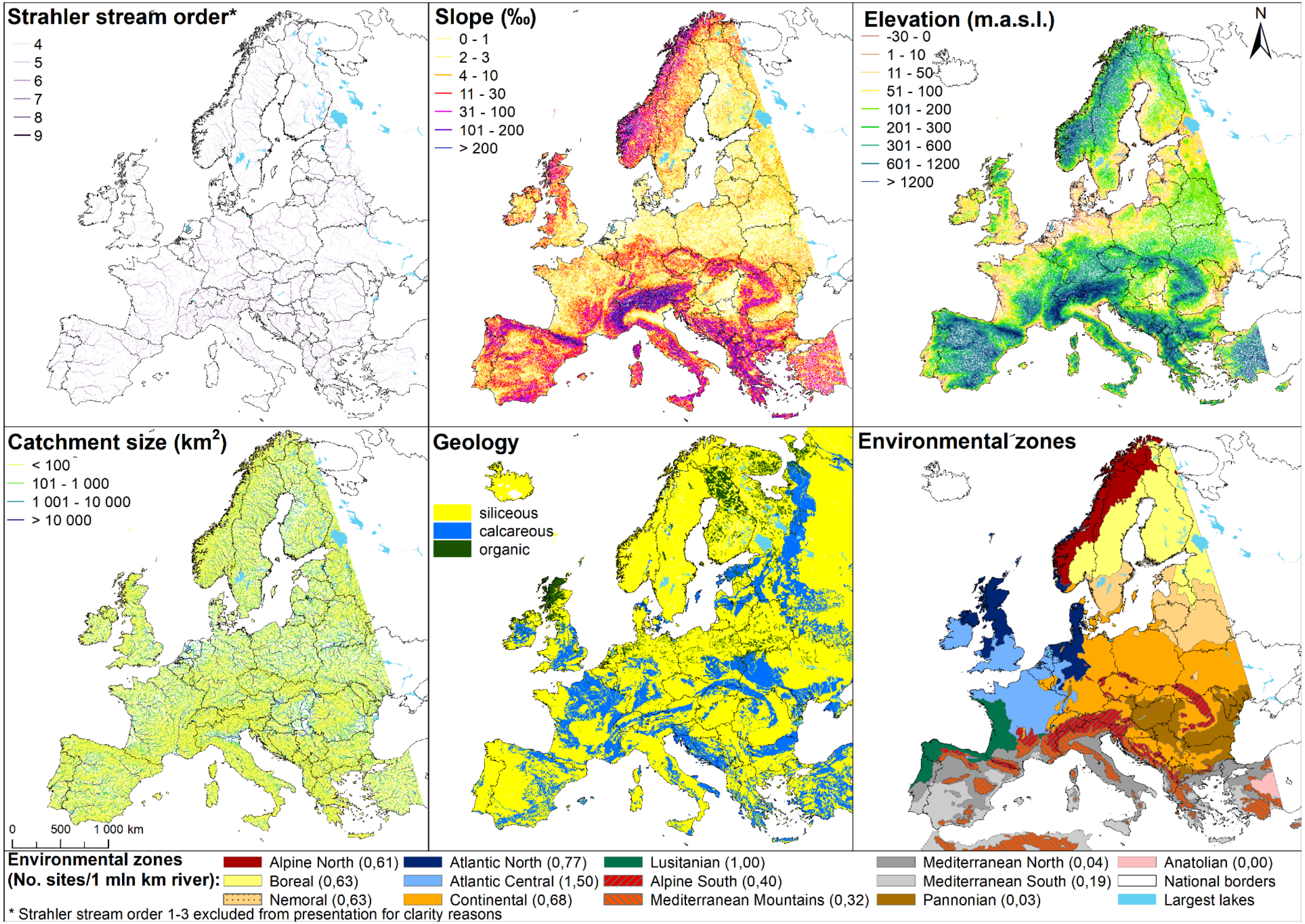

Supplementary Figure 1. **River segment's and catchment's characteristics used as proxies for delimitation of macrohabitat types (FCMacHTs) in European rivers.** Each panel refers to an individual attribute used as variable for modelling. River network (including slope, elevation, Strahler stream order, and catchment size) from CCM v2.1[33], geological classification from IHME 1500[34] and ESDB v 2.0[35] 35, environmental zones[36]. High resolution version of this figure is available[37].

Box 2. **Characteristics of the Fish Communities Macrohabitat Types (FCMacHT) based on broad environmental variables.**

Type 1. Pan-European calcareous headwater streams

This type distinguishes rivers and streams of highland, sub-mountain, upland and lowland regions characterized by calcareous bedrock distributed throughout central, western, and southern Europe. They exclude the Northern European Plain and Scandinavia. Altitudes are diversified, with 43% of rivers having an elevation below 300 m a.s.l. and 57% lying in the range of 300-2500 m a.s.l. The overall average is 457 m a.s.l. Rivers within this type are characterized by moderate (44%) to high (39%) slopes – 63 ‰ on average – and small catchments (93% are up to 50 km^2) composed of first and second order streams.

Fish communities associated with this habitat type consist mainly of intolerant highly rheophilic species (48%), with 24% rheophilic water column species and 12% rheophilic benthic species preferring sandy-gravel bottom substrate. These guilds are complemented by a moderate (16%) proportion of generalists. This particular fish assemblage typically occupy rivers with moderate to high slopes, cold, well aerated water, and medium or coarse bottom sediment.

Type 2. Mountain, Alpine rivers

This river type consists of streams and small rivers in mountainous areas across Central and Southern Europe, including the Alps, Carpathian, Sudeten, Pyrenees, and Cantabrian Mts., parts of Massif Central in France, the Dinaric Alps, and Balkan Mts. A majority (92%) of rivers are located within altitudes between 500 and over 3000 m a.s.l. (average 1164 m a.s.l.). Such rivers are characterized by very high slopes – 165‰ on average and more than 75% above 60‰. They are mainly first and second order streams (81%) within small catchments up to 50 km^2 (89%). Geology is mixed, either siliceous or calcareous.

Fish communities associated with this river type consist mainly of intolerant highly rheophilic species (55%), along with a 27% share of rheophilic water column species, and 18% rheophilic benthic species preferring sandy-gravel bottom substrate. This fish assemblage is typified by high slope rivers with cold, well-aerated water and coarse bottom sediment.

Type 3. Central European sub-mountain and lowland rivers

This type comprises Continental rivers and streams located mainly in Central and Eastern Europe in lowland areas from Germany, through Poland, to the Ukraine, as well as sub-mountain areas of the Carpathians, Sudeten, northern slopes of the Alpes, and Dinaric Alps, and intermountain regions of Bohemia, Moravia, Bavaria, and Ardennes. A majority of rivers (66%) are located at altitudes below 300 m a.s.l., with only 32.5% between 300 to 1500 m a.s.l. (overall average 256 m a.s.l.). These rivers are characterized by low slopes – 4‰ on average and 90% less than 10‰. This type excludes headwater streams with dominating second and third order rivers, and with 65% of them found in large, 50-1000 km^2 area catchments, having siliceous rock types.

The fish community of these rivers is composed of 37% rheophilic water column species preferring sandy-gravel bottom substrate, 19% intolerant highly rheophilic species, and 12% of rheophilic benthic species preferring sandy-gravel bottom substrate. Together, the four rheophilic fish guilds constitute 73% of the fish community, while two limnophilic guilds contribute 13%. Benthic species of moderate tolerance represent a small 5% share of the community, while generalists are moderately abundant with a 9% share. The high diversity of guild composition reflects the high habitat variability of provisioning rivers and streams distributed widely across this particular Continental bio-geographical region.

Type 4. Calcareous Alpine and highland rivers

Type 4 rivers complement Type 2 rivers of mountainous, Alpine regions in having lower-lying, less steep reaches of calcareous geology found in highlands from Central and Southern Europe. Most rivers (55%) are located in rugged areas at altitudes less than 1500 m a.s.l. (average 446 m a.s.l). Slopes are variable with 28% of rivers below 2‰ and 46% in the range 5-45‰, average 63‰. Rivers have a calcareous geology. Similarly to Type 3, headwater streams are excluded and second to forth order streams are dominating, with 89% located in large sized catchments (50-1000 km^2).

The fish community of these rivers is composed of 41% rheophilic water column species preferring sandy-gravel bottom substrate. Combined, all four rheophilic fish guilds constitute 75% of the fish community, while limnophilic phytophilic guild and benthic species of moderate tolerance guild contribute 8% and 7%, respectively. Generalists are moderately abundant with a 10% share of the fish community. Guild diversity is related to riverine habitat variability with a high representation of rheophilic species (similar to Type 3) due to the upland character of streams.

Type 5. Continental upland and lowland headwater streams

This type comprises Continental streams and small rivers located mainly in Central and Eastern Europe and the southern coast of Scandinavia in intermountain upland and lowland landscapes. Altitudes are mostly moderate to high, with 30% of rivers lying at altitudes 100-300 m a.s.l. and 53% at 300-1500 m a.s.l. Slopes are diversified, with 16% below 2‰ and 44% in the range of 5-45‰, average 42‰. The vast majority (97%) of rivers is located in small first and second order stream catchments and are not larger then sixth order with a siliceous geochemical composition.

The fish community of these rivers is composed of 39% intolerant highly rheophilic species. All four rheophilic fish guilds constitute 76% of the fish community, while two limnophilic guilds contribute to 14%. Generalists are moderately abundant with a 10% share in fish community. Strong domination of rheophilic guilds is connected to the more upland character of these rivers.

Type 6. Boreal lowland rivers

This river type group includes lowland and upland Boreal rivers and streams located in Scandinavia, Karelia and the northern Baltic Plains. Most rivers (92%) are located at altitudes below 500 m a.s.l. (average 195 m a.s.l.), with variable, mostly moderate slopes (majority below 45‰, average 10‰). These are mostly (88%) small catchments (up to 50 km^2) of first to third stream order not larger then seventh order.

The fish assemblage is dominated by three rheophilic guilds amounting for 68% of the fish community, with intolerant Highly rheophilic species being the most common (39%). The next most common guild – intolerant water column species, has a 13% share, while the two limnophilic species guilds together have a 13% share. Generalists are less common with only a 6% share. The fish community of this river type is quite complex, with six guilds present. Dominance of rheophilic guilds is connected to a higher occurrence of coarse bottom substrate in these rivers.

Type 7. Nemoral lowland rivers

This group consists of lowland Nemoral rivers located south of the Boreal region in southern part of Scandinavia and north-east of the Northern European Plains and small areas in the Polish Kashubia and Ukrainian Volyn regions. Most rivers (96%) are located in altitudes below 200 m a.s.l. (average 125 m a.s.l.) with low slope – a majority below 15‰, average 5‰. Catchments are small, with 67% up to 50 km^2 and comprised of first and second order streams not larger then seventh order. Geology is predominantly siliceous with some admixture of calcareous and organic substrate.

The fish assemblage is dominated by four rheophilic guilds that together account for 76% of the fish community, with dominance of intolerant highly rheophilic species (39%). Only one limnophilic guild has a 6% share, while benthic species of moderate tolerance have an 8% share. Generalists represent a modest 10% share. The fish community of this river type is quite complex, with six guilds present. Dominance of rheophilic guilds in these lowland rivers is connected to the more severe climate of the Nemoral zone and the frequent location of rivers in hilly, Lakeland landscapes with considerable ground water contribution.

Type 8. Mediterranean mountain and upland rivers

River type 8 comprises Mediterranean mountain and upland rivers. The majority of rivers (89%) are located at altitudes above 300 m a.s.l. with a maximum at 2 922 m a.s.l. and an average of 753 m a.s.l. River slopes are diversified from low to high (average 101‰). These rivers are distributed widely across the Mediterranean region from Portugal to Greece. Catchment substrates are partly siliceous and partly calcareous within small, first and second order streams.

Rheophilic guilds strongly dominate the fish community with a combined share of 91%. Within this group, intolerant highly rheophilic species are the most numerous (44%). Limnophilic lithophilic species of moderate tolerance make up 9% of fish community and generalists are absent. The high share of rheophilic guilds and a lack of generalists results from the mountainous character of the rivers in this type.

Type 9. South European plains and highland rivers

Rivers grouped in this type come from highland areas of the north Mediterranean, Pannonian, and Lower Danube regions, as well as highland parts of the Iberian Peninsula and western (Atlantic) part of France. These coastal and intermountain plain rivers are distributed widely across Southern Europe, from Western France, Portugal and Spain, through Italy and Greece, to the Pannonia and Black Sea coasts. River elevation is diversified from near sea level up to about 1000 m a.s.l., with an average elevation of 345 m a.s.l. River slopes are also diversified from low to high, average 42‰. Siliceous geology dominates.

Two guilds of rheophilic species are the most prevalent, with a common share of 58%. Limnophilic litophilic and limnophilic phytophilic species of moderate tolerance are also common, with 22% and 11%, respectively. The generalists share of the fish community is modest at 9%. The higher share of limnophilic

guilds, which are tolerant to higher temperatures and lower oxygen concentrations, in these highland rivers is connected to lower altitudes and the warmer climate conditions of South Europe.

Type 10. Mediterranean lowland rivers

This river type is distributed across the South Mediterranean region, especially in coastal zones, Mediterranean islands, and Southern Spain. Nearly all rivers (99.9%) are located below 1000 m a.s.l. and 65% are below 300 m a.s.l. Slopes are moderate, with 55% in the range 3-30‰ and 10% up to 1‰ (average 28‰ overall). Siliceous geology dominates. Catchments are mostly small in area (68% less than 50 km^2) and composed mainly of first and second order streams.

Limnophilic guilds are the most common in these rivers (48%), with 41% being limnophilic phytophilic species of moderate tolerance. Two guilds of rheophilic species (benthic and water column) together constitute 24%; highly rheophilic species are absent. Some benthic species of moderate tolerance (8%) can be found. The generalist share in fish community is relatively high at 20%. A high share of limnophilic guilds and generalists, which are tolerant to higher temperatures and lower oxygen concentrations, is connected with warm climate conditions and periodic droughts occurring in the South Mediterranean zone.

Type 11. Western European and Atlantic lowland rivers

This river type combines lowland and coastal streams of the Atlantic region in Ireland, England, North-central France, Belgium, the Netherlands, and western Germany. A majority of rivers (86%) are located in lowlands up to 300 m a.s.l., few (12%) up to 500 m a.s.l. (average 150 m a.s.l.). Slopes are typically low, averaging 12‰, with 90% below 30‰. Catchments tend to be less than 50 km^2 in area (69%) and mostly formed of first and second order streams (76%). Geological formations are mainly siliceous, with 30% of calcareous admixture.

The fish community is composed of only three guilds: intolerant highly rheophilic species and rheophilic benthic species, which make up 55% and 27% of the community, respectively, and a substantial 18% share of generalists. This peculiar fish assemblage is mainly linked to the relative variability of slopes as for lowland areas.

Type 12. Lowland organic rivers

This river type comprises lowland organic rivers and streams located mainly in central and eastern parts of the Northern European Plains and Scandinavia. Such rivers are distributed across Central Europe, from Austria, Switzerland and Germany, through Poland, to Ukraine, as well as Belarus, through Latvia, Estonia, and Russia to Finland, Sweden, and Norway, where catchments of organic substrate exist. Most rivers are located below 300 m a.s.l. (average 143 m a.s.l.) and characterized by low slope, with a majority below 10‰ (average 4‰). Most of catchments (75%) are less than 250 km^2 in size and 84% rivers are order three or lower.

The fish community of these rivers is highly diversified (nine guilds). It is composed mainly (66%) of rheophilic fish guilds, including 35% intolerant highly rheophilic species, 9% rheophilic benthic, and 18% water column species. The two limnophilic guilds contribute 10%. Benthic species of moderate tolerance represent 7% and intolerant water column species 5%. Generalists are quite abundant with a 12% share of the fish community. This complex guild structure reflects high habitat variability of rivers and streams distributed widely across the Continental and Boreal bio-geographical regions.

Type 13. Boreal mountain and highland rivers

River type 13 includes upland and mountain Boreal rivers and streams located in Norway and western parts of Sweden. A majority of rivers are situated at altitudes from 100 to 1000 m a.s.l. (average 465 m a.s.l.) with highly variable slope values – 21% of rivers have very low slopes no more than 3‰, the next 45% of river reaches have slopes ranging from 3 to 45‰, and 34% above 45‰. Most (75%) are located in small catchments less than 50 km^2 in area with first and second order streams being dominant, but not larger then sixth order streams.

The fish assemblage for this river type is quite specific, with a near majority (48%) of intolerant highly rheophilic intolerant species, a substantial share of rheophilic water column species (16%) and intolerant water column species (24%). The fourth guild present in rivers of this type are benthic species of moderate tolerance with a 12% share. Generalists are absent and all guilds found have high environmental specialization. Dominance of rheophilic and intolerant water column species guilds is connected to coarse bottom substrate in these rivers and the severe climatic conditions of the Alpine North zone.

Type 14. North Atlantic lowland and upland rivers

This type of river is distributed across Northern Ireland and central parts of the British Isles, Germany, and Denmark in the areas with dominant siliceous geology. It consists of coastal, lowland, highland, and low

mountainous rivers of the Northern Atlantic bio-geographical region. A majority of rivers (71%) are situated below 200 m a.s.l. and 29% are in the range 300-500 m a.s.l. (average 137 m a.s.l). River slopes are diversified, 18% of rivers have low slope (less than 1‰) and 52% range from 5 to 45‰ (average 19‰). These are rivers mainly (68%) located in medium sized catchments with an average area of 394 km^2. Over 75% of rivers are first and second order streams; none is not larger then seventh order.

Rheophilic groups dominate within these rivers, making up 76% of the fish community. Intolerant highly rheophilic species account for 48%, while rheophilic water column species and rheophilic benthic species account for 16% and 12%, respectively. The generalists share of the fish community is very high at 24%. The dominance of rheophilic guilds is connected to frequently pronounced slopes and coarse sediment, while the high share of generalists results from the lowland location of rivers.

Type 15. North Atlantic peatland rivers

The final river type is located in close proximity to type 14 rivers in Scotland and northern England, Netherlands, and Germany. A majority of rivers (51%) are elevated below 200 m a.s.l. and 41% have altitudes in the range 200-500 m a.s.l. (average 225 m a.s.l). River slopes are diversified, from low to high – 12% are less than 2‰ and 45% are in the range 5-45‰ (average 47‰). The catchments are mainly small with the vast majority (84%) covering less than 50 km^2 and largely (84%) made up of first and second order streams; all of them not larger then sixth order streams. A distinguishing feature of these rivers is that they flow through peatland areas, which is associated with organic bottom substrate.

Two rheophilic groups account for a large share (73%) of the fish community in these rivers, with 55% being intolerant highly rheophilic species and 18% rheophilic water column species. The generalists share of the fish community is the highest of any river type at 27%. Similarly to type 14 rivers, the dominance of rheophilic guilds is connected to considerable river slopes and the occurrence of coarse sediment, while the high share of generalists results from the lowland location of rivers and distinct organic geology.

3. Clustering and CART model

The figures below depict the details of the two-step cluster and discriminant analysis. Results of non-hierarchical analysis and Analysis of Group Similarities (ANOSIM) for the environmental attributes of undisturbed fish sampling sites is presented in Supplementary Figure 2, and the second step taking as variables guild proportions at undisturbed sites and site environmental cluster groupings obtained in the previous step in depicted in Supplementary Figure 3. Codes in R used for calculations are made available[38]. The resulting Classification and Regression Trees (CART) model is shown in Supplementary Figure 4. The ArcGIS toolbox and necessary data for calculating the FCMacHT codes for entire river network in provided[39].

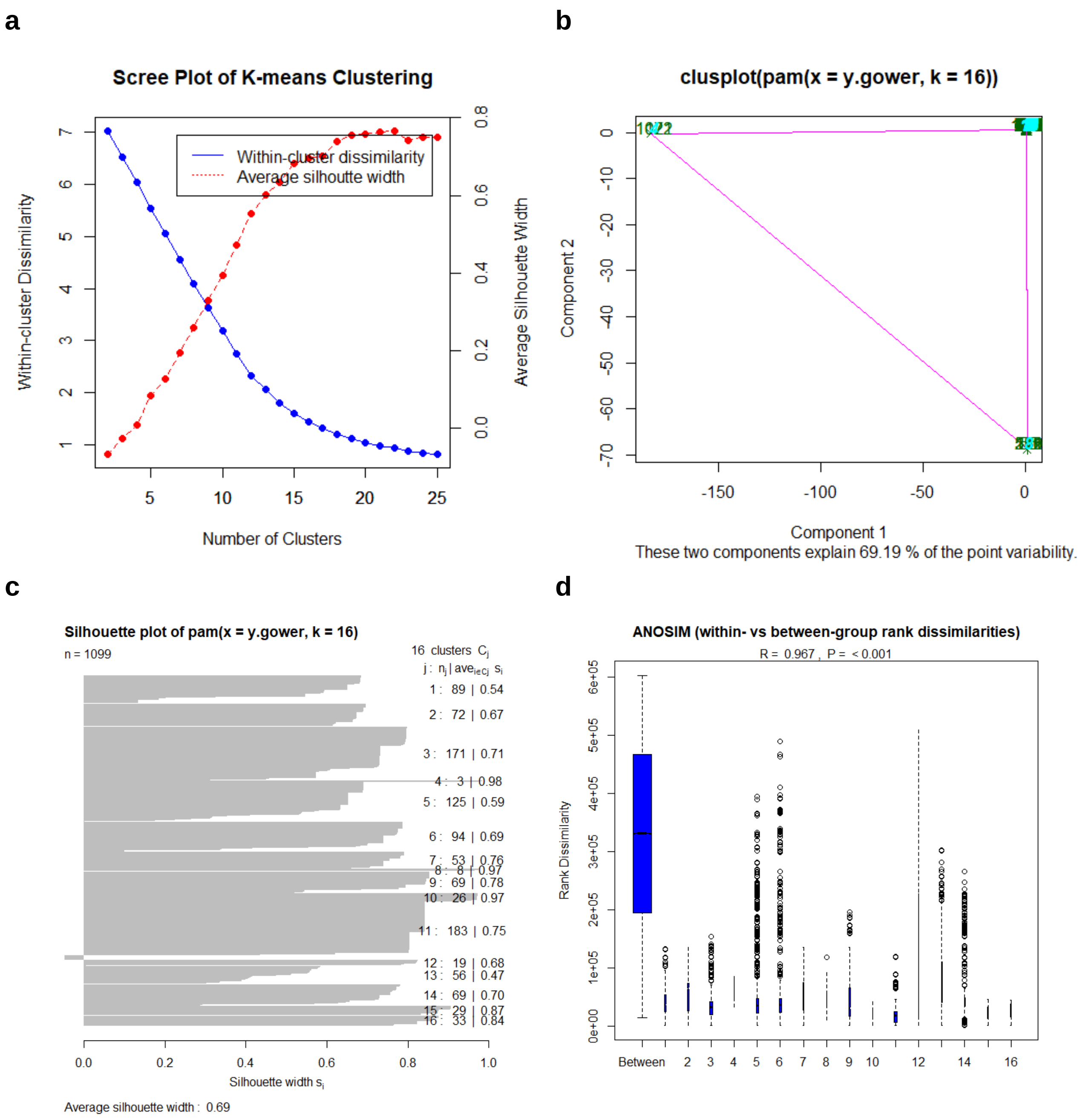

Supplementary Figure 2. **Results of non-hierarchical analysis and Analysis of Group Similarities (ANOSIM) for the environmental attributes of undisturbed fish sampling sites**. **a** Scree and silhouette plot. **b** Cluster for two main components with green elements denoting site numbers and pink lines the distances between ovoids. **c** Silhouette plot. **d** Box-and-whisker diagram showing distances between and within clusters according to ANOSIM test (R = 0.98, p = 0.001). Bounds of the box are defined as first and third quartile (Q1, Q3), center of the box represents median, whiskers represent minimum and maximum values limited to 1.5 times interquartile range (IQR). Circles represent outliers. Sample size n = 1099.

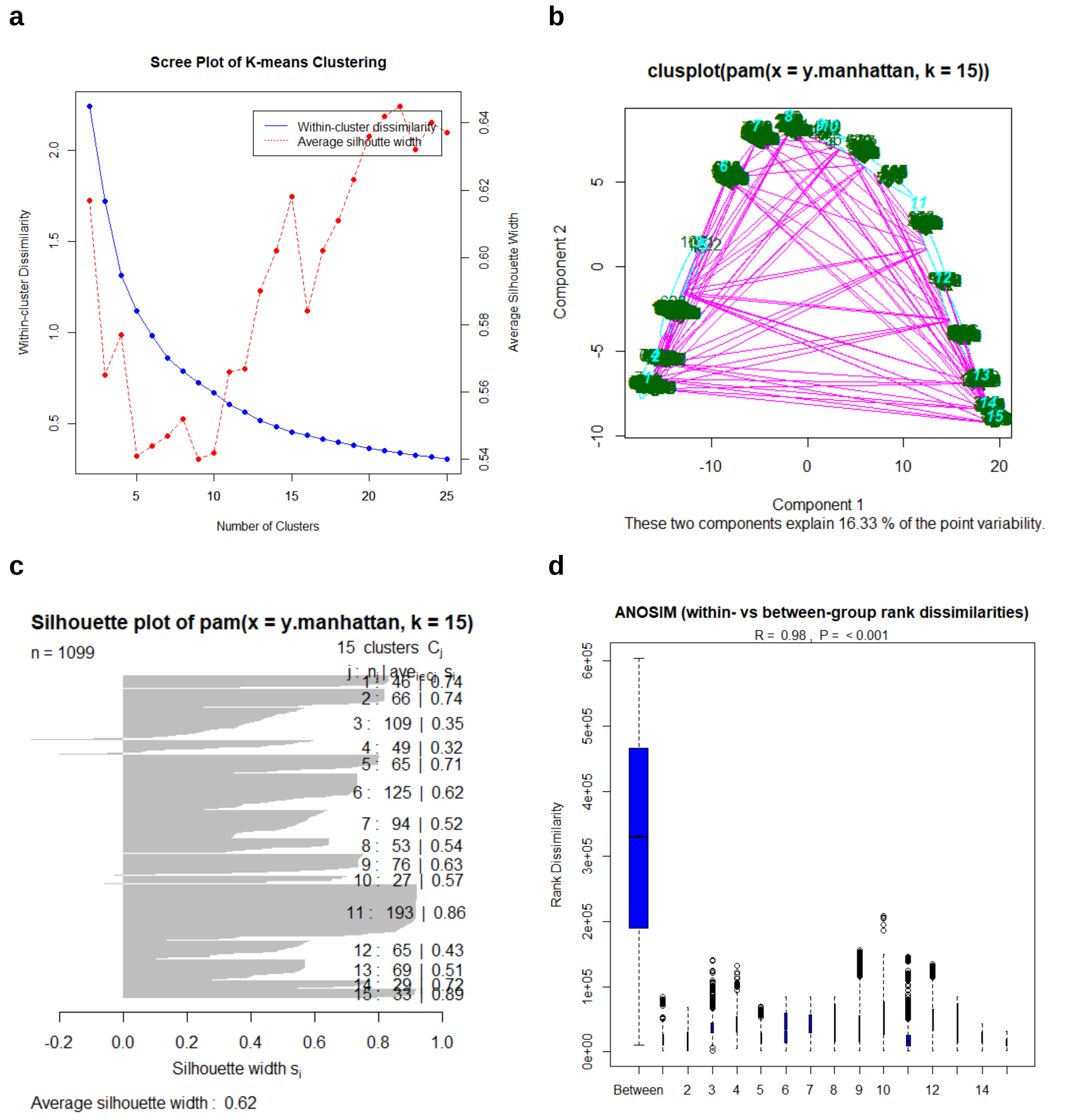

Supplementary Figure 3. **Results of non-hierarchical analysis and Analysis of Group Similarities (ANOSIM) of guild proportions at undisturbed sites and site environmental cluster groupings obtained in the previous step. a** Scree and silhouette plot. **b** Cluster for two main components with green elements denoting site numbers and pink lines distances between ovoids. **c** Silhouette plot. **d** Box-and-whisker diagram showing distances between and within clusters according to ANOSIM test (R = 0.98, p = 0.001). Bounds of the box are defined as first and third quartile (Q1, Q3), center of the box represents median, whiskers represent minimum and maximum values limited to 1.5 times interquartile range (IQR). Circles represent outliers. Sample size n = 1099.

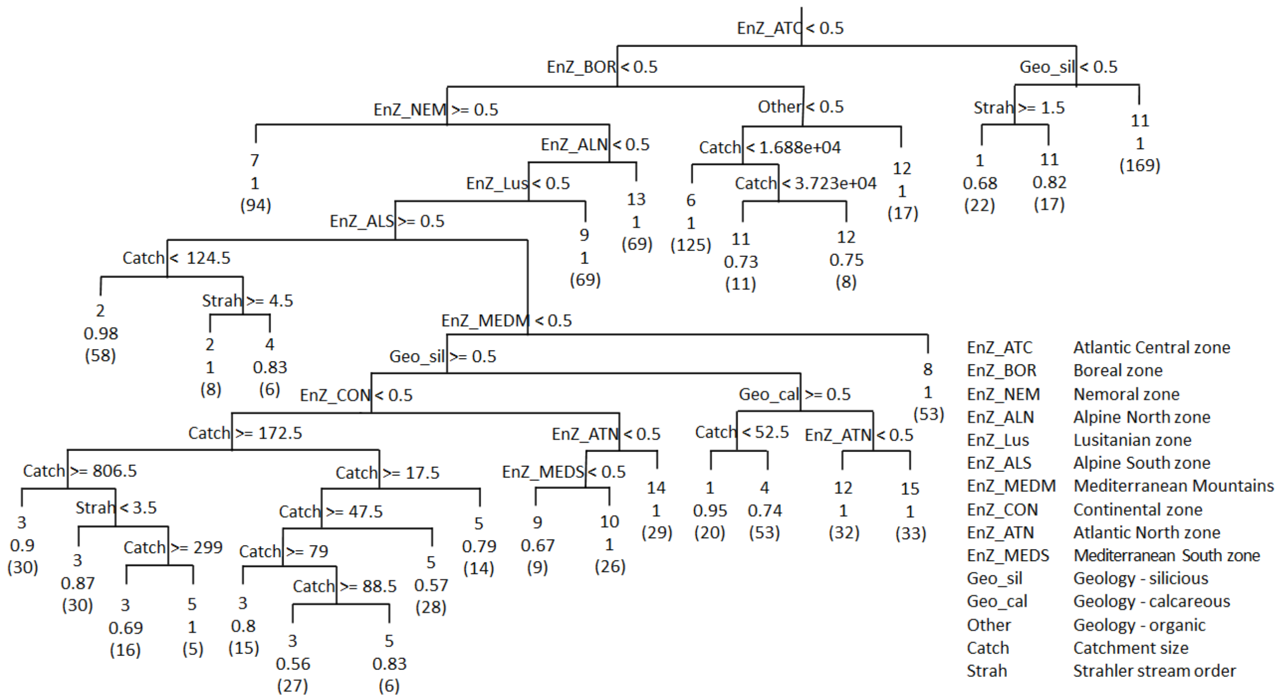

Supplementary Figure 4. **Classification and Regression Trees (CART) model.** Classification tree of environmental attributes according to FCMacHT classes. Kappa =0.86.

4. Additional discussion of impact assessment

The framework and models developed here were in part designed to meet the requirements of the ECOSTAT Working Group[40] by quantitatively defining the impact of barriers on fish communities and distinguishing between specific regions and barrier types. To accomplish this, we first determined the importance of various habitat attributes to each guild through definition of habitat preference (*HP*) indices (see Step 1. Calculation matrix provided as source dataset), followed by the definition of Habitat Alteration (*HA*) which reflect the relative loss or grain of specific habitat characteristic with respect to relative habitat for different barrier types (see Step 2. Calculation matrix provided as source dataset). Absolute loss or gain of habitat attributes was determined by taking the product of habitat preference and habitat alteration indices to produce a Habitat Suitability Alteration (*HSA*) score. An aggregate estimate for weighted remaining habitat proportion (*wHRp*) was derived by adding *HSA* scores, normalizing by habitat suitability and then weighting by the fish guild proportion (*GP*) within each FCMacHT (see Step 6. Calculation matrix provided as source dataset). It is worth noting that our approach does not take into account the technical / operational conditions of barriers nor their potential impacts on ecological potential. Table 1, therefore, roughly indicates how barrier impacts are differentiated among the different expected fish community types, but does not include all potential impacts on river habitat continuity and stability at the catchment scale. These omitted impacts can be grouped into the following two functional categories.

1. *Cumulative migration/connectivity impacts caused by reduced technical ability to pass fish at a given barrier and or reduced passability of barriers located downstream and upstream.* These factors cause disruption of migratory routes and, for anadromous fish species, in particular, can result in severe habitat loss and population decline whenever a single impassable barrier is present between their spawning grounds and the sea. The presence of barriers is also detrimental to potamodromous fish when feeding habitats are isolated from spawning areas.
2. *Alteration of habitat downstream caused by the operating condition of the barrier facility: hydropeaking, lack of environmental flows, or thermal alterations.* These factors influence both migratory and local fish species by rapidly changing water levels, modifying natural flow regimes, and physio-chemical water parameters. The extent and importance of such changes depends on the operational scheme used by barrier owners or the water management authority and sensitivity of individual species or guilds.

Figure 5 (main text) displays estimated median per barrier impacts across European catchments. It is important to note, however, that the European barrier inventory on which this analysis relies is incomplete, with a median underreporting error of 75% that varies considerably among countries. Identified issues include: whether a barrier has been recorded or not, whether its type is identified, and whether height information is available. Height underreporting error is about 45% and type underreporting is up to 65%[41]. Generally, the smaller a barrier is, the more probable it is that it has not been inventoried. This is cause for concern with regard to the accuracy of any overall barrier impact assessment, as undercounting of small barriers may significantly skew both projected barrier-level impacts and give an inaccurate perception regarding the overall habitat losses at the catchment scale. Indeed, in locations where small barriers are more routinely recorded (e.g., in Germany), barrier-level impacts within catchments estimated with median or average indicator will be more accurately anticipated to be low (indicated in blue in Figure 5). At the same time, this may be misleading in that even though small barriers typically only have a minor local impact, their cumulative impact may nonetheless be high due to the large number of them. Conversely, if small barriers are rarely recorded, then this will skew barrier-level impacts to be overly high because only large barriers are being assessed, while also mischaracterizing habitat loss at catchment scales as being high if and when the number/density of barriers is actually quite low.

Issues related to incomplete barrier data also have implications for impact management. Given that the majority of obstacles are unmapped, there is the real potential that river restoration efforts may be directed at high-cost projects that do little to address cumulative barrier effects. In short, there is a vital need for accurate and comprehensive mapping and inventorying of European barriers, including their impounding effects, as well as detailed technical guidance for remediating small barriers to maximize ecological benefits.

5. Data quality issues

Being a data driven procedure, any habitat classification and typology analysis is heavily dependent on the quality of input datasets. This is particularly true with regard to the fish catch dataset, including its accuracy and representativeness among undisturbed conditions across the different gradients of environmental variables, as well as the river network dataset and the set of environmental attributes derived for them. In addition, there are data quality issues related to our estimates of riverine habitat loss due to lack of knowledge about extant barrier impoundments and the necessity of making rough calculations based on incomplete barrier height data. Each of these points is discussed in turn below.

5.1. Fish catch data

Although the reference intercalibration (IC) dataset is the best available at the current time, it has some known limitations. For example criteria for reference conditions were not well defined for all countries at least for the first, pilot stage of the EU fish-based River Ecological Quality assessment[42]. Further, the research team was unable to obtain permission to use the data from several countries. While there is a central dataset maintained at the Joint Research Center, data copyrights belong to individual countries or (in case of Germany) individual administrative units. One country refused permission to use their data for any secondary analysis, though access rights have apparently been addressed by the ECOSTAT Working Group. Furthermore, some portions of Europe have very few undisturbed sites available for model calibration, while others have many (see Supplementary Figure 5). This resulted in uneven geographical distribution of the data used for our FCMacHT classification (see next subsection). It also needs to be pointed out that the IC data pertains to only one season (late summer) and to locations where fish migration may be limited by downstream barriers, hence some seasonal migratory fish or life stages may not be included in the FCMacHTs. At the time of selection for undisturbed sites by the ECOSTAT Working Group, the AMBER Barrier Atlas was not available. However, an ex-post analysis of the presence of downstream barriers contained in the AMBER Barrier Atlas for roughly 12% of undisturbed sites show that only about 69% of sites are free from an artificial obstacle below the surveyed river reach. Consequently, the fish assemblage recorded at many sampling sites might have been unsuspectingly affected by river discontinuity, thus altering the expected natural fish species composition. Last, but not least, the absence of any human pressures at survey sites should be taken with considerable caution. Despite these limitations, model performance is deemed to be very satisfactory and surpasses previous efforts

(Lek *et al.*, 2005). Expanding the initial set of undisturbed sites would naturally be expected to further improve model performance.

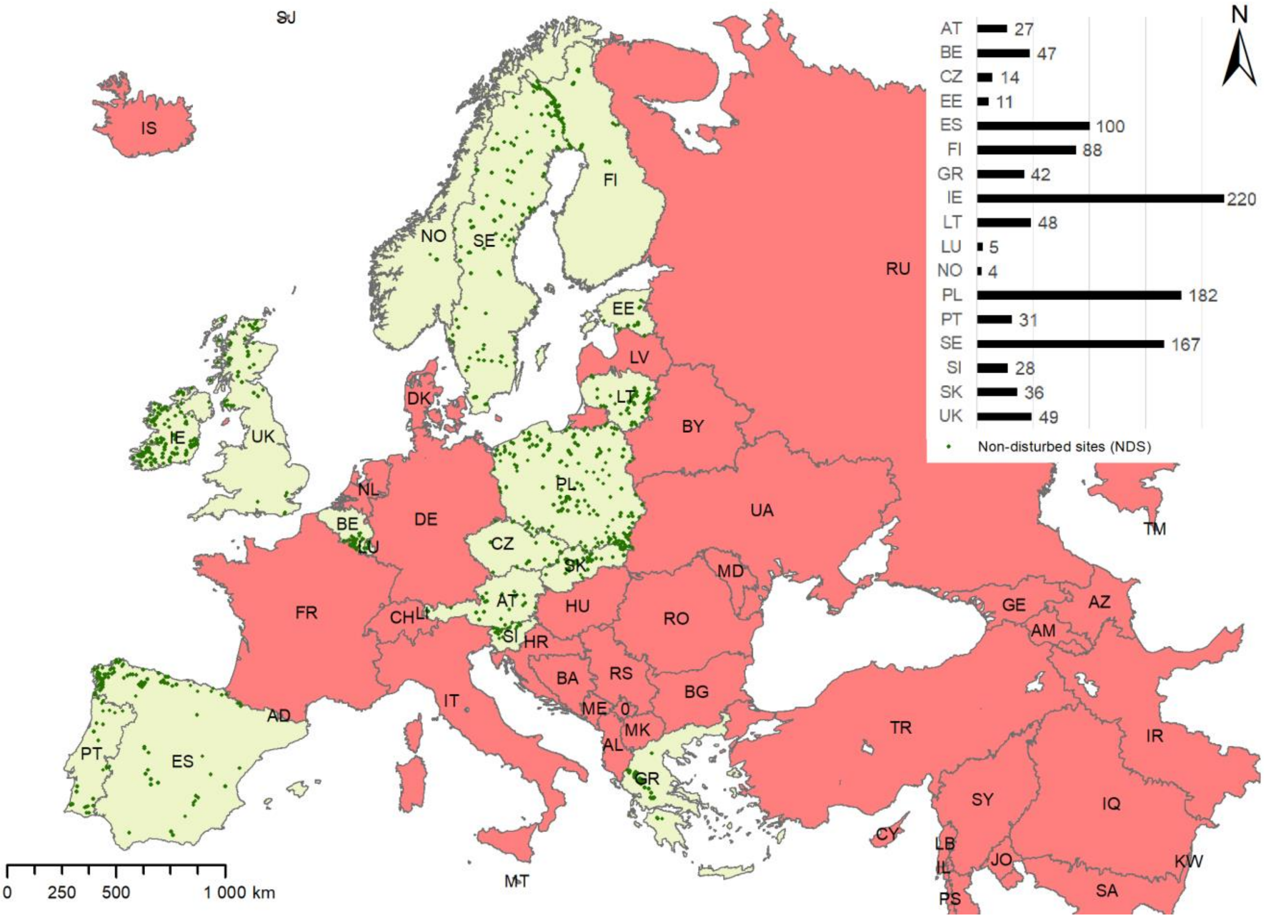

Supplementary Figure 5. **Location of undisturbed fishing sites from the Intercalibration (IC) dataset used for developing the macrohabitat typology (1099 sites).** Pale yellow – countries providing data; red – no permission to use IC data or a country not included in IC exercise; inset bar chart – number of sites per country.

5.2. Representativeness across environmental gradients

Good regional representation of undisturbed fish survey sites is important for accuracy of modeled fish communities. However, *de facto* representativeness of sampling sites differs considerably across geographical regions and environmental gradients. While low to moderate altitude and slope gradients are generally well represented, catchment sizes, river lengths, and Strahler stream order are clumped around relatively smaller values (Supplementary Table 3), presumably due to the greater ease of sampling smaller rivers. As such, large rivers and high mountain headwaters are not well covered, suggesting that the river classification may be less accurate in these locations.

The various environmental zones found across Europe[36,43] are mostly well represented by survey sites (see Supplementary Figure 1). That said, the number of sites from the Mediterranean, Anatolian, and Pannonian regions is comparatively smaller, while Iceland has no recorded fishing data available. By comparison, the Atlantic Central and Atlantic North zones are over represented with survey sites coming exclusively from Ireland and the British Islands. Continental IC fishing data were not available due to ownership issues described above. The Continental Zone is represented by a detailed Polish fish dataset, together with Czech, Slovakian, and Austrian data. The Alpine North zone is represented exclusively by fishing sites located on the eastern side of the Scandinavian mountains. Western, Atlantic-influenced sites are missing. The Lusitanian, Boreal, and Nemoral zones seem to be well represented across environmental zones. The South Alpine zone is represented by sites in the Alps, Sudeten, and eastern Carpathian Mountains, however, there is a lack of representation from the western Carpathian Mountains. A vast portion of the Pannonian zone is not covered by sampling sites nor is the Mediterranean North zone (see Supplementary Table 4).

Taking into account the strong environmental gradients occurring in Europe relative to the available reference dataset, the accuracy of results may be limited in some regions.

Supplementary Table 3. **Statistical summary of environmental attributes.** Results derived from the CCM v2.1 Rivers and Catchment database for undisturbed sites (training dataset) in comparison to the entire European river network (extrapolation dataset).

Environmental variables		Training dataset (n = 1096)						Extrapolation dataset (n = 851,820)					
	Unit	Min	Max	Mean	Median*	Mean deviation	Standard deviation	Min	Max	Mean	Median*	Mean deviation	Standard deviation
Strahler stream order number	-	1.0	6.0	-	2.0	-	-	1.0	9.0	-	1.0	-	-
Slope of the river segment	%	0.0	9.4	1.1	0.6	1.0	1.4	0.0	167.8	6.2	1.6	7.0	10.0
Mean elevation of a river segment	m a.s.l.	0	1437	256	166	196	261	-30	3305	509	348	381	484
Size of catchment upstream	km^2	1	40,112	1153	69	1783	4731	0	803,737	977	11	1727	13,609

*Mode for Strahler stream number

5.3. Broad environmental characteristics

The Catchment Characterization Model (CCM) dataset provides a consistent representation of hydrological networks and catchments for the entire European continent (Supplementary Figure 1). No better dataset of its kind was available at the time of the study. Nonetheless, the CCM, as well as the CCM-derived ECRINS dataset, suffer from geometric and spatial accuracy errors, especially in a low-relief areas[33,44,45]. This is clearly evident in the case of the middle reach of the Wda river in northwestern Lakeland, Poland (Supplementary Figure 6). The river course is inaccurately digitized, leading to incorrect geospatial attributes for length, Strahler stream order, and catchment size. As a result, some survey sites might have assigned much underestimated or overestimated values for these attributes. Consequently, fish communities typical for a middle reach of a medium-sized moraine river might have been assigned to a first order stream within a small catchment in such cases.

Comparison of CCM derived attributes and those obtained from the IC dataset related to environmental characteristics reveal that median error is 0.8% for altitude, 3.1% for catchment area, and 3.1% for slope. Mean values are significantly higher at 12.9%, 134.6%, and 104.3%, respectively (see Supplementary Table 5). This is mainly due to very high percent errors for a small set of undisturbed sites, which could not be corrected, despite numerous attempts. For geological classification, for 69% of sites CCM and IC classifications agree. Inconsistency likely stems from both inaccuracies in the CCM dataset, as well as inaccurate recording of site environmental characteristics by field survey teams. In CCM, slope has been measured along a river segment defined as a length of river between two confluences, while in the IC dataset the following EFI+ provision is used: “the stream segment should be as close as possible to 1 km for small streams, 5 km for intermediate streams and 10 km for large streams (Data source: maps with scale 1:50 000 or 1:100 000)”[3,46]. Similarly, altitude values in CCM correspond to the mean altitude of a river segment’s beginning and the end. For the IC dataset, these values are collected at site level. The CCM river network slopes range from 0 to 167.8%, which exceeds by more than 8 times the upper limit for slope in the IC database. However slopes above 20% only constitute about 2.3% of the total river network length and are mainly associated with small, first order mountainous streams.

Supplementary Table 4. **Statistical summary of environmental attributes for each FCMacHT.** Similarity level calculated for European Environmental Zones of Europe used as one of the variables. ALN – Alpine North, BOR – Boreal, NEM – Nemoral, ATN – Atlantic North, ATC – Atlantic Central, CON – Continental, LUS – Lusitanian, ALS – Alpine South, MDM – Mediterranean Mountains, MDN – Mediterranean North, MDS - Mediterranean South, PAN – Pannonian, ANA – Anatolian.

		**FCMacHT**															**Total**
		**1**	**2**	**3**	**4**	**5**	**6**	**7**	**8**	**9**	**10**	**11**	**12**	**13**	**14**	**15**	
**Strahler stream order**	**Min**	1	1	1	1	1	1	1	1	1	1	1	1	1	1	1	**1**
	**Mode**	1	1	3	4	1	1	1	1	1	1	1	1	1	1	1	**1**
	**Max**	8	7	9	9	5	7	7	8	9	8	8	9	6	7	6	**9**
**Slope (‰)**	**Min**	-	-	-	-	-	-	-	-	-	-	-	-	-	-	-	**-**
	**Max**	1632	1678	299	710	758	660	170	1150	750	795	426	331	1111	720	549	**1678**
	**Mean**	63	165	4	12	42	10	5	101	42	28	12	4	57	19	47	**62**
	**SD**	84	147	9	21	62	23	7	101	62	43	22	9	91	32	58	**100**
**Altitude (m a.s.l.)**	**Min**	-7	-3	-10	-5	-9	-4	-6	-4	-8	-6	-6	-8	-8	-5	-30	**-30**
	**Max**	2128	3305	1748	1886	2,376	1332	354	2922	2113	1406	1037	1197	2001	744	908	**3305**
	**Mean**	457	1164	256	446	426	195	125	753	345	250	150	143	465	144	225	**509**
	**SD**	358	531	199	343	355	192	56	371	291	197	134	126	325	123	167	**484**
**Size of catchment up-stream (km2)**	**Min**	-	-	48	53	-	1	-	-	-	-	-	-	-	-	1	**-**
	**Max**	71,882	13,444	574,773	802,633	298	16,877	101,767	186,341	802,660	96,619	160,620	803,737	11,670	143,656	6056	**803,737**
	**Sum**	5,234,628	12,313,615	179,727,078	64,493,360	1,042,973	31,603,597	59,069,985	15,081,156	287,605,373	46,324,731	65,676,622	48,027,214	8,788,104	7,158,633	392,944	**832,540,013**
	**Mean**	61	85	6338	2371	16	407	1139	150	2560	1325	1928	3112	184	394	45	**977**
	**SD**	608	515	28,794	31,664	30	1604	6375	2301	28,141	7131	9974	22,732	791	3994	185	**13,609**
**Length of river by geological type (km)**	**Siliceous**	-	95,556	107,777	6261	148,663	203,647	110,063	94,558	295,490	107,563	79,820	1087	108,275	51,790	-	**1,410,549**
	**Calcareous**	195,423	75,312	-	71,380	-	14,389	23,556	68,982	18,626	-	29,380	185	1914	-	-	**499,148**
	**Organic**	3220	597	-	73	-	-	15,003	-	658	-	2408	72,480	2914	-	20,562	**117,914**
**Sum of river length by Environmental Zones (km)**	**ALN**						0							113,103			**113,103**
	**BOR**						218,036					2464	36,680				**257,180**
	**NEM**							148,623									**148,623**
	**ATN**	5848			2619	2									51,790	20,562	**80,822**
	**ATC**	29,551										109,141					**138,692**
	**CON**	76,256		107,777	33,612	148,661			1			1	34,466				**400,775**
	**LUS**									70,461							**70,461**
	**ALS**		171,465		9866												**181,332**
	**MDM**								163,538								**163,538**
	**MDN**	39,160			12,459					148,735	1		408				**200,762**
	**MDS**	25,478			6221						107,562						**139,260**
	**PAN**	18,395			10,976					88,142			2198				**119,711**
	**ANA**	3954			1961					7437							**13,351**
**FCMacHT similarity to EnZ (%)**		38	100	100	43	100	100	100	100	47	100	98	50	100	100	100	**20**
**Length of river network (km)**		**198,642**	**171,465**	**107,777**	**77,714**	**148,663**	**218,036**	**148,623**	**163,540**	**314,774**	**107,563**	**111,607**	**73,751**	**113,103**	**51,790**	**20,562**	**2,027,611**

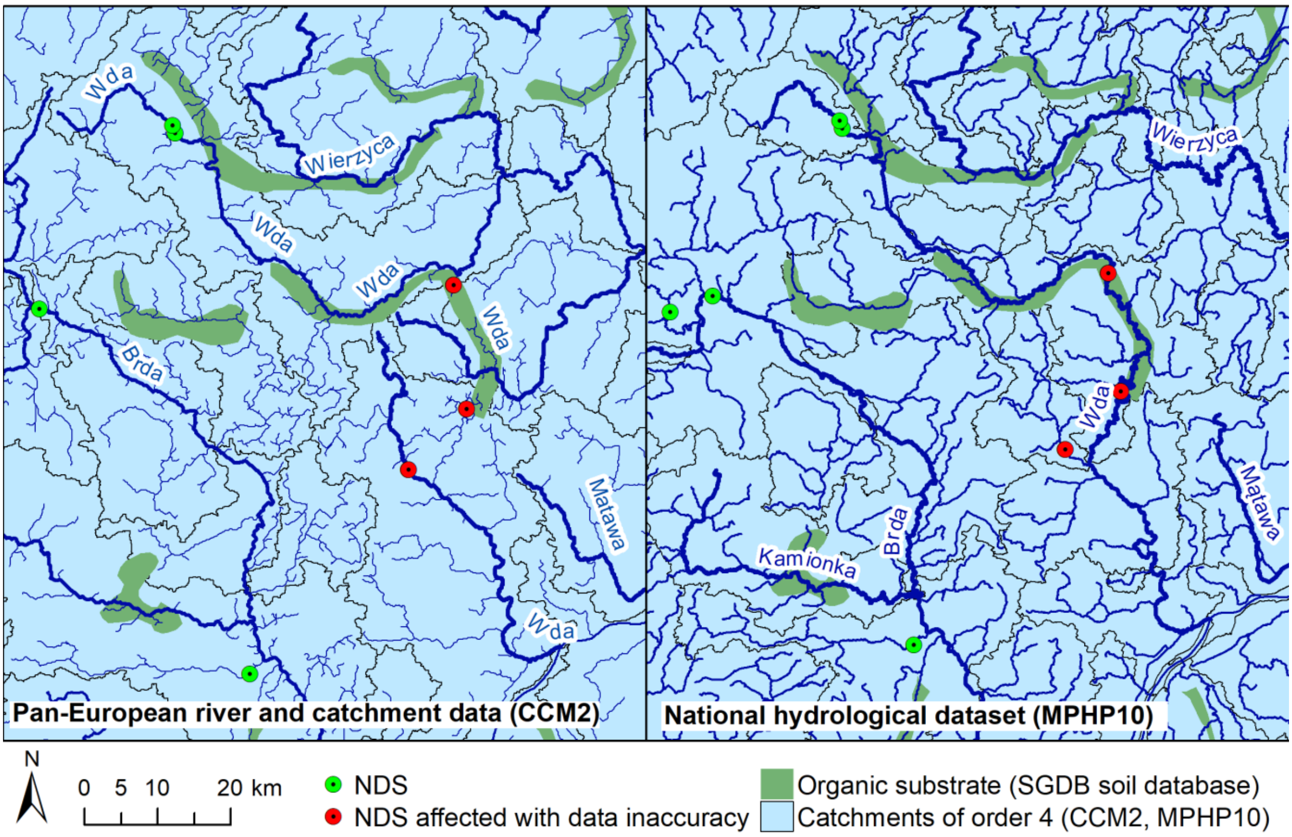

Supplementary Figure 6. **Example of geometric accuracy and precision issues with the CCM river network.** This rises potential consequences for ecological and habitat modelling. Example from north-western Lakeland, Poland. Left: CCM v2.1 River and Catchment Database (CCM2); Right: reference dataset of Mapa Podziału Hydrograficznego Polski 1:10,000 (MPHP10), National Water Management Authority Wody Polskie. Organic substrate derived from European Soil Database, v2.0.

Supplementary Table 5. **Percent error for environmental variables at undisturbed sites.** Statistics obtained from CCM v2.1 River and Catchments database relative to those obtained from a IC fish monitoring dataset.

Statistic	Altitude error (%)	Catchment size error (%)	Slope error (%)
Mean	12.9	134.6	104.3
Median	0.8	3.1	3.1
Standard deviation	77.1	2431.9	427.9
Mean deviation	28.8	241.9	186.6

Evident errors with the CCM may have also been created through a combination of the coarse resolution of the digital elevation model (100 m) and the use of an automated procedure to obtain height values for stream segments at starting and ending points. It is likely that some height values might have been erroneously obtained from a valley slope instead of the river bed in the CCM product. Indeed, there are cases where the river reach starting point height is lower than the end point. In addition, some undisturbed sites are likely to have been incorrectly assigned to a river reach due to geometric inaccuracies of the river network. To help avoid this, where it was not possible to precisely locate an undisturbed site due to global positioning system precision issues, the site was excluded from our analysis. The identification (and possible correction) of all mislocated rivers and catchments present in CCM, even just for the subset of undisturbed sites, was beyond permissible time and effort. Correction of the CCM / ECRINS datasets, as envisioned in their documentation[45], is eagerly anticipated and would allow for a revised and more accurate macrohabitat classification and barrier impact assessment. We believe, however, that for a broad brush, regional scale analysis, the available data are sufficiently accurate.

In the EFI+ procedure, geology is assigned to one of three classes: siliceous, calcareous, or organic based on the dominant category[3]. Here, the method for assigning river geological type accounts for local characteristic of geology within the direct local catchment area and disregards geological characteristics found upstream. The work of Lyche Solheim et al.[47] accounts for dominate geology of the wider catchment, which we believe would be useful to consider as part of any future version of our proposed habitat typology.

Tanneberger et al.[48] recently compiled a European map of peatlands. However, this information was not available to us in a digital format (geospatial database). Our analysis, based on the Soil Geographical Database of Eurasia (SGDBE)[49], may underrepresented peatlands compared to Tanneberger et al.[48], especially in part of Scandinavia, Central Europe, and southern parts of Europe, though a relatively good approximation of organic substrate was available for the northern part of British Islands, north Finland, and the northeastern part of Europe (Russia).

Perhaps the biggest issue around environmental data quality is related to soils, and to organic soils in particular. The European Soil Database v2.0 (SGDBE) used to derive organic substrate in this study was created at a very coarse 1:1,000,000 scale [50]. Overall positional accuracy of the dataset has been estimated to between 500-5000 m with minimum area covered of 9 ha. There are also some known generalization issues affecting the accuracy of soil locations[49]. These include necessary data aggregation (Soil Typological Units were aggregated into Soil Mapping Units), potentially leading to inaccuracies in spatial representation and a dominating value of thematic information. The soil layers also lack border harmonization for some countries and there are some discrepancies in the amount of detail given for object geometry and/or description. On top of this, not only were data collected over wide range of dates, with the earliest data recorded in 1980, the latest recorded in 1996[49], but imprecisions on the location of particular organic soils have been introduced as a result of the generalization procedures applied. For the reasons listed above, in some regions organic-depended fish habitat may not be sufficiently accurate. Nevertheless, the above dataset was the best available. To classify areas with organic substrate, three soil attributes (soil type, dominant soil parent material, dominant surface textural class) were used (see Supplementary Table 6).

Supplementary Table 6. **Geological formation classification rules.**

Geochemical category	Rules for classification	Data source*
Formations classified as siliceous	Lithological units identified as: Fine sediments (except those with marls), coarse sediments (except those with marls), siliciclastic rocks and fine sediments, siliciclastic rocks and coarse sediments, siliciclastic rocks, metamorphic rocks (except of those with marbles and limestones), magmatic rocks (except of those with limestones)	IMHE1500
Formations classified as calcareous	Lithological units identified as: Fine sediments (with marls), coarse sediments (with marls and limestones), calcareous rocs, calcareous rocks and coarse sediments, calcareous rocks and fine sediments, metamorphic rocks (with marbles and limestones), metamorphic rocks (marbles)	IMHE1500
Organic substrate	Soil mapping units classified as: marsh or Histosol according to the World Reference Base (WRB) for Soil Resources, organic materials or peat (mires) according to dominant soil parent material, peat soils according to Dominant surface textural class.	ESDB v2.0

* IHME1500 – International Hydrogeological Map of Europe (IHME 1500, v1.2); ESDB v2.0 – European Soil Database v2.0.

Last but not least, we found 85% overall similarity of FCMacHT groupings with the environmental zones derived from the Environmental Stratification of Europe[36] indicating that this aggregate variable was the most influential for the results.

5.4. Impoundment length calculation

Impoundment length was calculated only for sites where barrier height was available. We used a simplistic Pythagorean formula for determining impoundment length, which likely underestimates

true impoundment length in most cases. In typical engineering practice, backwater depths are computed iteratively for each upstream cross-section until water depth differs ± 1-2 cm from normal water depth. The distance from the structure to this particular cross-section is called the backwater length. Alternatively, backwater length is also defined as the point upstream at which elevated water depth is within 1% of the normal water depth[51].

When designing or assessing hydraulic structures, calculations of the backwater depth and length usually involve the use of different equations that require detailed knowledge of various flow and physical parameters, including a geometric profile of each cross-section and channel roughness and slope. Where this is not feasible, Rühlmann's formula designed to use for channels with a rectangular cross-section, or Tolkmitt's formula – for channels with parabolic cross-sections, are sometimes used in engineering practice to obtain rough estimates for backwater length[52].

Rühlmann's formula expresses backwater length L_R as:

$$L_R = \frac{d_n}{s} \cdot f_R\left(\frac{\Delta h}{d_n}\right) \qquad (S1)$$

where d_n represents normal, unobstructed water depth, s the bottom slope, and f_R is the value read from a table for a specialized function of the ratio of added water depth Δh to normal depth d_n.

Alternatively, Tolkmitt's formula calculates backwater length L_T as follows:

$$L_T = \frac{d_n}{s} \cdot f_T\left(\frac{d_n+\Delta h}{d_n}\right) \qquad (S2)$$

with values of the function f_T also obtained in tabular form.

The example backflow length estimates using Rühlmann's and Tolkmitt's formulas are presented in Supplementary Table 7. The estimated backwater lengths given the same parameters clearly differ, with Rühlmann's estimates are always greater than Tolkmitt's.

In our study, we used the following approximation for backwater length, denoted $\hat{L}$:

$$\hat{L} = \frac{h}{s} \qquad (S3)$$

where h is the height of a barrier measured from its base and so corresponds to $h + \Delta h$ using the above notation. We observe that our estimate for impoundment length (with Equation S3) falls between the two values obtained with Rühlmann's (Equation S1) and Tolkmitt's formulas (Equation S2).

The barrier height data were used to obtain impoundment length for the available CCM river network. Owing to the way in which river segments are defined within the database (confluence bounded river sections) it is possible that some barriers were attributed to the segments that were shorter than the estimated impoundment lengths formed by those barriers. In most cases, the river segment's gradient was greater than the local gradients (i.e., of nearby segments), thus resulting in shorter estimated impoundment length than would be expected in reality. This fact further lends credence to our estimates of impoundment length being very conservative.

Supplementary Table 7. **Comparative results of computing backwater length.** Different formulas were tested to derive the impoundment length: Rühlmann's formula (L_R), Tolkmitt's formula (L_T), and the formula used within this study ($\hat{L}$). d_n – normal depth, s – slope of river reach, Δh – water depth.

No	d_n [m]	s [-]	Δh [m]	L_R [m]	L_T [m]	$\hat{L}$ [m]
1	1.0	0.0002	0.5	8561	6692	7500
2	1.5	0.0002	1.0	14,169	11,276	12,500
3	2.0	0.0002	1.2	18,183	14,385	16,000
4	1.0	0.0005	0.5	3424	2677	3000
5	1.5	0.0005	1.0	5667	4511	5000
6	2.0	0.0005	1.2	7273	5754	6400
7	1.0	0.0010	0.5	1712	1338	1500
8	1.5	0.0010	1.0	2834	2255	2500
9	2.0	0.0010	1.2	3637	2877	3200

6. Supplementary references

1. Pont, D. *et al.* Assessing river biotic condition at a continental scale: A European approach using functional metrics and fish assemblages. *Journal of Applied Ecology* **43**, 70–80 (2006).
2. Melcher, A., Schmutz, S., Haidvogl, G. & Moder, K. Spatially based methods to assess the ecological status of European fish assemblage types. *Fish Manag Ecol* **14**, 453–463 (2007).
3. EFI+ Consortium. *Manual for the application of the new European Fish Index-EFI+. A Fish-Based Method to Assess the Ecological Status of European Running Waters in Support of the Water Framework Directive*. (2009).
4. Fausch, K. D., Lyons, J., Karr, J. R. & Angermeier, P. L. Fish communities as indicators of environmental degradation. *American Fisheries Society Symposium* **8**, 123–144 (1990).
5. Harrison, T. D. & Whitfield, A. K. A multi-metric fish index to assess the environmental condition of estuaries. *J Fish Biol* **65**, 683–710 (2004).
6. Segurado, P. *et al.* Estimating species tolerance to human perturbation: Expert judgment versus empirical approaches. *Ecol Indic* **11**, 1623–1635 (2011).
7. Dahm, V. *et al.* Effects of physico-chemistry, land use and hydromorphology on three riverine organism groups: A comparative analysis with monitoring data from Germany and Austria. *Hydrobiologia* **704**, 389–415 (2013).
8. Logez, M., Bady, P. & Pont, D. Modelling the habitat requirement of riverine fish species at the European scale: sensitivity to temperature and precipitation and associated uncertainty. *Ecol Freshw Fish* **21**, 266–282 (2012).
9. Karr, J. R. Assessment of biotic integrity using fish communities. *Fisheries (Bethesda)* **6**, 21–27 (1981).
10. Schinegger, R., Trautwein, C., Melcher, A. & Schmutz, S. Multiple human pressures and their spatial patterns in European running waters. *Water and Environment Journal* **26**, 261–273 (2012).
11. McCabe, D. J. Rivers and Streams: Life in Flowing Water. *Nature Education Knowledge* **1**, 1–14 (2010).
12. Brooks, E. G. E., Freyhof, J. & Brooks, E. *European Red List of Freshwater Fishes*. (Publications Office of the European Union, 2011). doi:https://doi.org/10.2779/85903.
13. Parasiewicz, P. *et al. D2.2 Conceptual Model of Ecological Impacts of Barriers in EU Considering Fish Habitat Selection Criteria for Running Waters*. (2019).
14. Parasiewicz, P. Using Mesohabsim to develop reference habitat template and ecological management scenarios. *River Res Appl* **23**, 924–932 (2007).
15. Forio, M. A. E. *et al.* Fuzzy modelling to identify key drivers of ecological water quality to support decision and policy making. *Environ Sci Policy* **68**, 58–68 (2017).
16. Poff, N. L. R. & Ward, J. V. Physical habitat template of lotic systems: Recovery in the context of historical pattern of spatiotemporal heterogeneity. *Environ Manage* **14**, 629–645 (1990).
17. Welcomme, R. L., Winemiller, K. O. & Cowx, I. G. Fish environmental guilds as a tool for assessment of ecological condition of rivers. *River Res Appl* **22**, 377–396 (2006).
18. Parasiewicz, P., Prus, P., Suska, K. & Marcinkowski, P. “E = mc2” of Environmental Flows: A Conceptual Framework for Establishing a Fish-Biological Foundation for a Regionally Applicable Environmental Low-Flow Formula. *Water (Basel)* **10**, (2018).
19. Jungwirth, M., Muhar, S. & Schmutz, S. Re-establishing and assessing ecological integrity in riverine landscapes. *Freshw Biol* **47**, 867–887 (2002).
20. Wolter, C., Bischoff, A. & Wysujack, K. The use of historical data to characterize fish-faunistic reference conditions for large lowland rivers in northern Germany. *Large Rivers* **15**, 37–51 (2003).
21. McKenna, JE, J. *et al.* A broadscale fish-habitat model development process, Genesee Basin, New York. in *Symposium on Landscape Influences on Stream Habitats and Biological Assemblages, American Fisheries Society, Symposium 48* (2006).
22. Steen, P. J., Zorn, T. G., Seelbach, P. W. & Schaeffer, J. S. Classification Tree Models for Predicting Distributions of Michigan Stream Fish from Landscape Variables. *Trans Am Fish Soc* **137**, 976–996 (2008).
23. Lyons, J., Stewart, J. S. & Mitro, M. Predicted effects of climate warming on the distribution of 50 stream fishes in Wisconsin, U.S.A. *J Fish Biol* **77**, 1867–1898 (2010).

24. McKenna, J. E. & Castiglione, C. Hierarchical multi-scale classification of nearshore aquatic habitats of the Great Lakes: Western Lake Erie. *J Great Lakes Res* **36**, 757–771 (2010).
25. McKenna, J. E., Ruggirello, J. E. & Johnson, J. H. A landscape-based distribution model for fallfish (Semotilus corporalis) in the Great Lakes drainage of New York. *J Great Lakes Res* **38**, 413–417 (2012).
26. *Modelling Community Structure in Freshwater Ecosystems*. (Springer Berlin Heidelberg, 2005). doi:10.1007/b138251.
27. Fausch, K. D., Torgersen, C. E., Baxter, C. v. & Li, H. W. Landscapes to riverscapes: Bridging the gap between research and conservation of stream fishes. *Bioscience* **52**, 483–498 (2002).
28. Mckenna, J. E., Schaeffer, J. S., Stewart, J. S. & Slattery, M. T. Development of a spatially universal framework for classifying stream assemblages with application to conservation planning for great lakes lotic fish communities. *Restor Ecol* **23**, 167–178 (2015).
29. Jowett, I. G. & Duncan, M. J. Flow variability in new zealand rivers and its relationship to in-stream habitat and biota. *N Z J Mar Freshwater Res* **24**, 305–317 (1990).
30. Verzano, K. *et al.* Modeling variable river flow velocity on continental scale: Current situation and climate change impacts in Europe. *J Hydrol (Amst)* **424–425**, 238–251 (2012).
31. Bjerkjlie, D. & Sturtevant, L. *Simulated Hydrologic Response to Climate Change During the 21st Century in New Hampshire Scientific Investigations Report 2017 – 5143*. (2018).
32. Guse, B. *et al.* Eco-hydrologic model cascades: Simulating land use and climate change impacts on hydrology, hydraulics and habitats for fish and macroinvertebrates. *Science of the Total Environment* **533**, 542–556 (2015).
33. De Jager, A. & Vogt, J. *Rivers and Catchments of Europe - Catchment Characterisation Model (CCM). [dataset]* PID: http://data.europa.eu/89h/fe1878e8-7541-4c66-8453-afdae7469221 (2007).
34. BGR & UNESCO. *International Hydrogeological Map of Europe 1:1,500,000 (IHME1500). Digital map data v1.2. [dataset]*. *[dataset]* https://produktcenter.bgr.de/terraCatalog/DetailResult.do?fileIdentifier=341255A9-180F-4BF9-B96F-D085339EA86D (2019).
35. European Commision & European Soil Bureau Network. *The European Soil Database distribution version 2.0, CD-ROM*. *EUR 19945 EN* vol. EUR 19945 EN https://esdac.jrc.ec.europa.eu/content/european-soil-database-v20-vector-and-attribute-data (2004).
36. Metzger, M. J. The Environmental Stratification of Europe. *[dataset]* (2018).
37. Parasiewicz, P. *et al.* Over 200,000 kilometers of free-flowing river habitat in Europe is altered due to impoundments. *figshare* (2023) doi:https://doi.org/10.6084/m9.figshare.22730897.
38. Parasiewicz, P. & McGarigal, K. Over 200,000 kilometers of free-flowing river habitat in Europe is altered due to impoundments. FCMacHT clusters calculation codes: Publication release. *Zenodo* (2023) doi:10.5281/ZENODO.8004302.
39. Belka, K. Over 200,000 kilometers of free-flowing river habitat in Europe is altered due to impoundments. Classification and Regression Tree model for calculating FCMacHT codes. *Zenodo* (2023) doi:10.5281/ZENODO.8001121.
40. Halleraker, J. H. *et al. Working Group ECOSTAT report on common understanding of using mitigation measures for reaching Good Ecological Potential for heavily modified water bodies. Part 1: Impacted by water storage*. *Hydropower Status Report* (Publications Office of the European Union, 2016). doi:10.2760/722208.
41. Belletti, B. *et al.* More than one million barriers fragment Europe's rivers. *Nature* **588**, 436–441 (2020).
42. Jepsen, N. & Pont, Didier. *Intercalibration of fish-based methods to evaluate river ecological quality: report from an EU intercalibration pilot exercise.* (Office for Official Publications of the European Communities, 2007).
43. Metzger, M. J., Bunce, R. G. H., Jongman, R. H. G., Mücher, C. A. & Watkins, J. W. A climatic stratification of the environment of Europe. *Global Ecology and Biogeography* **14**, 549–563 (2005).
44. de Jager, A. L. & Vogt, J. V. Development and demonstration of a structured hydrological feature coding system for Europe. *Hydrological Sciences Journal* **55**, 661–675 (2010).

45. European Environment Agency. *European catchments and Rivers network system (Ecrins). Version 1*. *[dataset]* https://www.eea.europa.eu/data-and-maps/data/european-catchments-and-rivers-network (2012).
46. Schinegger, R., Melcher, A. & Schmutz, S. *EFI+ D3.1 Report on hydromorphological pressures D3.2 Global pressure index*. (2008).
47. Lyche Solheim, A. *et al.* A new broad typology for rivers and lakes in Europe: Development and application for large-scale environmental assessments. *Science of the Total Environment* **697**, 134043 (2019).
48. Tanneberger, F. *et al.* The peatland map of Europe. *Mires and Peat* **19**, 1–17 (2017).
49. Soil Geographical Database of Eurasia at scale 1:000,000 version 4 beta. [dataset]. in *The European Soil Database distribution version 2.0, CD-ROM* vol. EUR 19945 EN (European Commission, 2004).
50. CEC. *Soil map of the European Communities at 1:1,000,000*. (CEC-DGVI, 1985).
51. Czetwertyński, E. *Hydraulika i hydromechanika*. (PWN, 1985).
52. Skibiński, J. *Hydraulika*. (PWN, 1969).
