# Supplementary material for: Over 200,000 kilometers of free-flowing river habitat in Europe is altered due to impoundments
Source: Nat Commun. 2023 Oct 9;14:6289. doi: 10.1038/s41467-023-40922-6 (PMC10562483; doi:10.1038/s41467-023-40922-6)
Supplement: Supplementary file 2 — Description of Additional Supplementary Files [file 41467_2023_40922_MOESM2_ESM.pdf]

Description of Additional Supplementary Files

File name: Supplementary Data 1.xlsx
Description: Details for calculation of weighted Riverine Habitat proportion (*wRHp*, Step 2) and *FCMacHT sensitivity* (Step 5) indices.
