# Supplementary material for: Over 200,000 kilometers of free-flowing river habitat in Europe is altered due to impoundments
Source: Nat Commun. 2023 Oct 9;14:6289. doi: 10.1038/s41467-023-40922-6 (PMC10562483; doi:10.1038/s41467-023-40922-6)
Supplement: Supplementary file 4 — Reporting Summary [file 41467_2023_40922_MOESM4_ESM.pdf]

nature portfolio

Corresponding author(s): Piotr Parasiewicz

Last updated by author(s): May 8, 2023

Reporting Summary

Nature Portfolio wishes to improve the reproducibility of the work that we publish. This form provides structure for consistency and transparency in reporting. For further information on Nature Portfolio policies, see our Editorial Policies and the Editorial Policy Checklist.

Statistics

For all statistical analyses, confirm that the following items are present in the figure legend, table legend, main text, or Methods section.

n/a	Confirmed	
☐	☒	The exact sample size (n) for each experimental group/condition, given as a discrete number and unit of measurement
☐	☒	A statement on whether measurements were taken from distinct samples or whether the same sample was measured repeatedly
☐	☒	The statistical test(s) used AND whether they are one- or two-sided<br>*Only common tests should be described solely by name; describe more complex techniques in the Methods section.*
☒	☐	A description of all covariates tested
☐	☒	A description of any assumptions or corrections, such as tests of normality and adjustment for multiple comparisons
☐	☒	A full description of the statistical parameters including central tendency (e.g. means) or other basic estimates (e.g. regression coefficient) AND variation (e.g. standard deviation) or associated estimates of uncertainty (e.g. confidence intervals)
☒	☐	For null hypothesis testing, the test statistic (e.g. F, t, r) with confidence intervals, effect sizes, degrees of freedom and P value noted<br>*Give P values as exact values whenever suitable.*
☒	☐	For Bayesian analysis, information on the choice of priors and Markov chain Monte Carlo settings
☒	☐	For hierarchical and complex designs, identification of the appropriate level for tests and full reporting of outcomes
☒	☐	Estimates of effect sizes (e.g. Cohen's d, Pearson's r), indicating how they were calculated

Our web collection on statistics for biologists contains articles on many of the points above.

Software and code

Policy information about availability of computer code

Data collection	no specific software was used
Data analysis	ESRI 2011. ArcGIS Desktop: Release 10. Redlands, CA: Environmental Systems Research Institute, R ver. 3.2 The R Foundation (packages:boot, energy, MASS, vegan, nortest, energy, rpart, cluster, fpc and custom algorthims: mvstarts.R, cartware.R by Kevin McGarigal, Clus_FCMacHT_EnZ.R by Parasiewicz), Microsoft Office 2013,

For manuscripts utilizing custom algorithms or software that are central to the research but not yet described in published literature, software must be made available to editors and reviewers. We strongly encourage code deposition in a community repository (e.g. GitHub). See the Nature Portfolio guidelines for submitting code & software for further information.

Data

Policy information about availability of data

All manuscripts must include a data availability statement. This statement should provide the following information, where applicable:

- Accession codes, unique identifiers, or web links for publicly available datasets
- A description of any restrictions on data availability
- For clinical datasets or third party data, please ensure that the statement adheres to our policy

Fish catch data and location of non-disturbed sites were obtained from EU fish-based River Ecological Quality assessment referenced here as intercalibration dataset (IC). While there is a central dataset maintained at the Joint Research Center, data copyrights belong to individual countries or (in case of Germany) individual administrative units. We obtained written permission to reuse the data from individual entities with no further right to republish the data. Access to these data requires a written request to JRC, contact person Wouter van de Bund (Wouter.VAN-DE-BUND@ec.europa.eu). More information at https://www.nffa.eu/about/consortium/site/?id=43 . The broad environmental characteristics of the river sites and non-disturbed sites were acquired from the River and Catchment

Database derived from the Catchment Characterization Model (CCM2.1)[43], the International Hydrogeological Map of Europe (IHME1500, v1.2) at a scale of 1:500,000[44], European Soil Database (ESDB, v2.0)[4763], the Environmental Stratification of Europe[48]. The variables derived from these sources and used for the analysis are made available under a CC-BY-4.0 license here: https://doi.org/10.6084/m9.figshare.22730897 [64]. Barrier data (Fig. 6) as well as the underlying data for Table 2 come from the AMBER Barrier Atlas and are freely available at https://amber.international/european-barrier-atlas/ under a CC-BY-4.0 license and here: https://doi.org/10.6084/m9.figshare.12629051.v5 [65]. Data used for generation of Fish Community Macrohabitat Types map (Fig. 3), fish sensitivity to impounding map (Fig. 4), estimated barrier habitat impact across European rivers with respect to barrier type (Fig. 5), projected barrier-level impounding impacts in European catchments (Fig. 6), and river segment's and catchment's characteristics used as proxies for delimitation of macrohabitat types (FCMacHTs) in European rivers (Supplementary Fig. 1) are made available under a CC-BY-4.0 license here: https://doi.org/10.6084/m9.figshare.22730897 [64].

Field-specific reporting

Please select the one below that is the best fit for your research. If you are not sure, read the appropriate sections before making your selection.

☐ Life sciences ☐ Behavioural & social sciences ☒ Ecological, evolutionary & environmental sciences

For a reference copy of the document with all sections, see nature.com/documents/nr-reporting-summary-flat.pdf

Ecological, evolutionary & environmental sciences study design

All studies must disclose on these points even when the disclosure is negative.

Study description	This ecology – environmental study is based on the analysis of data obtained from published and freely available databases containing fish biological information as well as databases containing description of broad environmental attributes, such as topography, river network characteristics, geology and soil, and bio-climatic zones. These data were used to define expected fish-macrohabitat distribution in rivers to serve as a benchmark for determination of impact of barriers on fish habitat. First, fish species were grouped into guilds according to their known behaviors, then data from 1099 non-disturbed sites describing habitat attributes together with observed guilds abundance were non-hierarchically clustered. For each cluster expected habitat structure is defined using expected guild proportions in a local fish community as a proxy. It is calculated applying biocomplexity theory derived model to relative fish abundance in a sample. Subsequently, Classification and Regression Trees (CART) analysis is applied to classify all rivers in Europe. The impact of barrier impoundment in specific location is derived from barrier type and local macrohabitat structure.<br>The quantiative data on fish captures were transferred into relative distributions per site. The quantitative characteristics of river sections such as Strahler stream order, catchment area, altitude, slope and geological type were untreated and potential redundancies and interaction with environmental zones were investigated by analyzing model performance. Since slope and altitude did neither improved the model nor changed clustering results these two variables were removed from the final clustering procedure.
Research sample	The fish biological data was gathered by European Commission's Working Group on Ecological Status (ECOSTAT) during river monitoring intercalibration exercise. The broad environmental characteristics of the river sites and non-disturbed sites were acquired from River and Catchment Database derived from the Catchment Characterization Model (CCM2.1), the International Hydrogeological Map of Europe (IHME1500, v1.2) at a scale of 1:500,000, European Soil Database (ESDB, v2.0), the Environmental Stratification of Europe. We also used data from European Barrier Atlas providing information about the type and location of barriers on European Rivers. This research sample represented all data available for the study from European countries. It combines biological and non-biological data to provide a comprehensive picture of expected habitats. Intercalibration data was limited by obtained permitions and licenses to use.
Sampling strategy	The input sample size was maximized to the available intercalibrated fish dataset that fulfilled the study criteria. The fish biological data set selected for this study consisted of 1099 samples, which represented rivers in non-distrurbed conditions in majority of European landscape forms. Therefore it was sufficient for the statistical analysis and model calibration. The analysis involved calculation of expected fish habitat structure in a river section is also based on the available dataset.<br>To compute expected fish community for specific river type a set of 10 sites were selected at random from each cluster and used to calculated the expected guild proportions in the community. The number of the sample sites is based on the metaanalysis of publicly available Target Fish Community reports from rivers (n=22) in New Hampshire (https://www.des.nh.gov/resource-center/publications?keys=tfcrpt). In these studies n was defined using Multivariate Pseudo Standard Error (MultSE51) and only rarely exceeded n=10.<br>The data used for model extrapolation consisted of broad characteristics for 851821 river sections representing majority of the rivers in the European Union.
Data collection	We used existing datasets (fish data, broad environmental characteristics). The fish biological data included in intercalibration data base was collected with electrofishing equipment by scientists and agencies from members countries. The sampling approach was according to the standard EN 14011; CEN, 2003.
Timing and spatial scale	Fish sampling data included in the intercallibration database took place between 2001 and 2017 throughout a year. Study refers to European territory.
Data exclusions	The input database was filtered to select sites that fulfill the criteria of being non-disturbed or little disturbed by human actions. The criteria of non-disturbance were defined in the database and unified across countries during the intercalibration process. Many of rivers in Europe are impacted by human induced alterations and fish community structure is reflecting that. Therefore to establish a benchmark for impact determination we had to exclude the samples where we expect to have modified fish community composition. Also for site descriptors we excluded attributes that are frequently modifiable by human actions (e.g. sinuosity). Some sampling sites fulfilling the near-naturalness criteria had to be excluded from the analysis due to the proprietary rights, others did not have proper geographic location and had to be excluded.

Reproducibility	The clustering procedure was repeated 5 times and provided consistent results. The only aspect of our analysis that may vary with successive trials are the results of random subdivision of sets and selection of samples for defining expected fish community. To keep this variability under control, the draws were repeated 3 times, unless no more than 10 samples were available.
Randomization	Excels' pseudorandom number generator was used to select sites for determining expected fish community structure. The samples were selected at random.
Blinding	Blinding was not relevant because we used the existing and reviewed database, and we did not use subjective assessments in the course of the analysis.

Did the study involve field work? ☐ Yes ☒ No

Reporting for specific materials, systems and methods

We require information from authors about some types of materials, experimental systems and methods used in many studies. Here, indicate whether each material, system or method listed is relevant to your study. If you are not sure if a list item applies to your research, read the appropriate section before selecting a response.

Materials & experimental systems

n/a	Involved in the study
☒	☐ Antibodies
☒	☐ Eukaryotic cell lines
☒	☐ Palaeontology and archaeology
☒	☐ Animals and other organisms
☒	☐ Human research participants
☒	☐ Clinical data
☒	☐ Dual use research of concern

Methods

n/a	Involved in the study
☒	☐ ChIP-seq
☒	☐ Flow cytometry
☒	☐ MRI-based neuroimaging
